# Apolipoprotein E Alleles Across the Spectrum of Frontotemporal Lobar Degeneration: A Systematic Review and Meta-Analysis

**DOI:** 10.3390/ijms27177752

**Published:** 2026-08-29

**Authors:** Ioannis Liampas, Antonis Polyviou, Silvia Demiri, Gerasimos Malataras, Vasilis Oikonomakis, Vasileios Siokas, Odysseas Kargiotis, Zinovia-Maria Kefalopoulou, Elisabeth Chroni, Efthimios Dardiotis

**Affiliations:** 1 Department of Neurology, School of Medicine, University of Thessaly, 41500 Larissa, Greece; vsiokas@med.uth.gr (V.S.); edar@med.uth.gr (E.D.); 2 Department of Neurology, School of Medicine, University of Patras, 26504 Rio Patras, Greece; kargiody@gmail.com (O.K.); zkefalopoulou@gmail.com (Z.-M.K.); echroni@upatras.gr (E.C.); 3 School of Medicine, University of Patras, 26504 Rio Patras, Greece; polyviouantonis@gmail.com (A.P.); sylviantemiri@gmail.com (S.D.); malatarasgerasimos@gmail.com (G.M.); 4 Department of Psychiatry, University Hospital of Patras, 26504 Rio Patras, Greece; vasilisoikonomakis@gmail.com

**Keywords:** progressive supranuclear palsy, frontotemporal dementia, corticobasal syndrome, corticobasal degeneration, motor neuron disease

## Abstract

We conducted a systematic review and meta-analysis of associations between *apolipoprotein E* (*APOE*) alleles and frontotemporal lobar degeneration (FTLD)-spectrum disorders. MEDLINE, Embase, CENTRAL, and Google Scholar were searched. *APOE2* and *APOE4* carrier status were compared between FTLD-spectrum disorders and healthy controls (HCs) or individuals with Alzheimer’s disease (AD). Forty studies were included. *APOE4* carriage was more frequent in frontotemporal dementia (FTD) compared with HC (OR = 1.72; 95% CI = 1.45–2.04) and less common than in AD (OR = 0.35; 95% CI = 0.29–0.42). In contrast, *APOE2* carriage was less prevalent in FTD relative to HC (OR = 0.83; 95% CI = 0.70–0.98) but more frequent compared with AD (OR = 1.80; 95% CI = 1.29–2.52). *APOE4* effects were most pronounced in behavioral variant FTD. In clinically confirmed progressive supranuclear palsy (PSP), *APOE4* carriage was not associated with PSP. Analysis restricted to pathologically confirmed PSP cases, however, showed lower *APOE4* carriage in PSP than in healthy controls (OR = 0.78, 95% CI = 0.65–0.94), although this association failed to reach the multiplicity-adjusted significance threshold. *APOE2* carriage was not associated with PSP in either clinically established or pathologically confirmed samples. Evidence was insufficient to establish or exclude associations for other FTLD-spectrum disorders because of the limited available data. In conclusion, *APOE* alleles show distinct associations across the FTLD spectrum.

## 1. Introduction

Apolipoprotein E (APOE), encoded by the *APOE* gene, is a major lipid-transport protein in the central nervous system and one of the most well-established genetic modifiers of neurodegenerative disease [1,2]. The three common *APOE* alleles—*APOE* ε2 *(APOE2), APOE* ε3 *(APOE3),* and *APOE* ε4 *(APOE4)*—show markedly different associations with Alzheimer’s disease (AD). *APOE4* represents the strongest common genetic risk factor for sporadic late-onset AD, with a dose-dependent effect on disease risk, whereas *APOE2* is considered the strongest common protective allele [2,3,4]. Given their central role in AD, increasing focus has been placed on the association between *APOE* and susceptibility across the spectrum of frontotemporal lobar degeneration (FTLD). This growing interest aligns with a broader shift in modern neurology toward deep phenotyping, in which disease characterization extends beyond syndromic presentation to incorporate detailed clinical, imaging, genetic, and molecular features [5,6].

FTLD is a neuropathologic umbrella term referring to a group of neurodegenerative disorders characterized predominantly by degeneration of the frontal and anterior temporal lobes. The principal clinical syndromes associated with FTLD are collectively referred to as frontotemporal dementia (FTD) and include behavioral variant FTD (bvFTD), semantic variant primary progressive aphasia (svPPA), and nonfluent/agrammatic variant primary progressive aphasia (nfvPPA) [7,8]. The broader FTLD spectrum also encompasses corticobasal syndrome (CBS), progressive supranuclear palsy (PSP), and FTD with motor neuron disease (FTD-MND), reflecting the substantial clinical and pathological overlap between these disorders and the core FTD syndromes [9,10]. At the molecular level, FTLD is also heterogeneous, with major pathological subtypes including FTLD-tau and FTLD-TDP [11,12,13]. In contrast to AD, the relationship between *APOE* and FTLD remains uncertain. Over the past several decades, association studies have produced conflicting findings, likely reflecting the considerable clinical and neuropathologic heterogeneity within FTLD [14,15,16]. Analyses that pool phenotypically and pathologically diverse patient groups may obscure subtype-specific genetic effects.

A clearer understanding of the relationship between *APOE* and FTLD may provide important insights beyond risk estimation alone. Such knowledge could contribute to improved understanding of the pathophysiological mechanisms underlying specific clinical subgroups, refinement of disease classification, and the development of targeted therapeutic strategies [15,17]. We therefore systematically evaluated *APOE2* and *APOE4* carriage across FTLD-spectrum phenotypes relative to both healthy controls and individuals with AD, enabling assessment of associations relative to normal ageing and to a neurodegenerative disorder strongly influenced by *APOE*.

## 2. Methods

The current systematic review and meta-analysis conforms with the Preferred Reporting Items for Systematic reviews and Meta-Analyses (PRISMA) [18] and Meta-analysis Of Observational Studies in Epidemiology (MOOSE) reporting guidelines [19]. It was registered in the online database of PROSPERO prior to its initiation (registration number: CRD420261323774). All study procedures were independently performed by two authors (A.P. and S.D.). Discrepancies were resolved by a third author (I.L.).

### 2.1. Search Strategy

MEDLINE (via PubMed), Embase, and CENTRAL were systematically searched from inception through January 2026, with Google Scholar used as a supplementary search source. The search was restricted to article titles for Google scholar and to article titles, abstracts and keywords for Embase and CENTRAL. For PubMed, the following search strategy was used (adapted as appropriate for the other databases):

*((((((((((((((((frontotemporal dementia) OR (frontotemporal lobar degeneration)) OR (FTD)) OR (FTLD)) OR (bvFTD)) OR (primary progressive aphasia)) OR (PPA)) OR (agrammatic aphasia)) OR (nonfluent aphasia)) OR (semantic dementia)) OR (FTLD TDP)) OR (FTLD-tau)) OR (corticobasal syndrome)) OR (corticobasal degeneration)) OR (progressive supranuclear palsy))) AND (apolipoprotein)*.

The reference lists of all retrieved articles, as well as relevant systematic reviews and meta-analyses, were manually screened to identify further eligible studies (backward snowballing). Forward citation tracking of the included studies was also performed through PubMed. All titles and abstracts were screened for eligibility. Full texts of potentially relevant articles were subsequently retrieved to determine whether they met the predefined inclusion criteria.

### 2.2. Eligibility Criteria

Studies were included according to the following criteria:

- Published from database inception through January 2026.
- Designed as case–control, cross-sectional, or cohort studies.
- Including adult participants with FTLD spectrum disorders. For case–control and cross-sectional studies, comparison groups were required to include adults with AD and/or cognitively unimpaired HC. For cohort studies, the relative risk of incident FTLD spectrum disorders by *APOE* status (allele carriage or specific genotype) had to be estimated in cognitively unimpaired individuals.
- Reporting *APOE* genotype frequencies and/or *APOE2* and/or *APOE4* carriage status.
- Clinical diagnoses were established by expert clinicians according to internationally accepted diagnostic criteria applicable at the time the study was conducted.

Exclusion criteria were as follows:

- Cognitive diagnoses not based on internationally accepted clinical diagnostic criteria. For studies comparing FTLD with AD, studies were excluded if AD diagnosis was not established according to internationally accepted clinical diagnostic criteria. For studies comparing FTLD with HC, the presence of other neurodegenerative disorders among HC constituted an exclusion criterion.
- Mixed neurodegenerative cohorts without separable subgroup data.
- Duplicate cohorts; in such cases, the largest or most complete dataset was included.
- Lack of data on *APOE* genotype and/or *APOE2*/*APOE4* carriage status.
- Other study designs (e.g., reviews, meta-analyses, case reports).
- Short communications without detailed methods to establish eligibility and methodological rigor.
- Participant groups with ≤15 participants (to minimize variability and ensure robustness of statistical estimates).
- Full texts and abstracts not published in English.

### 2.3. Quality Assessment Tool

Case–control studies were assessed using the Newcastle–Ottawa Scale (NOS), which evaluates key methodological aspects and their reporting quality. The NOS was slightly modified to better reflect the specific features of this study. The full assessment tool is provided in Table S1. Cross-sectional studies were treated analytically as analogous to case–control studies, as *APOE* allele carriage was compared between predefined diagnostic groups and comparator groups at a given time point. Accordingly, these studies were assessed using the same modified case–control Newcastle–Ottawa Scale framework to ensure consistent evaluation of comparable methodological domains. No cohort studies were ultimately included in the meta-analysis.

### 2.4. Data Extraction and Outcome Measures

The following data were extracted according to standardized data extraction forms: first author, year of publication, study design, settings and country of origin, ancestry/ethnicity/population background, APOE genotyping method, diagnostic criteria, clinical/neuropathological confirmation, number of participants, age and sex distribution, disease duration and MMSE scores. We also obtained *APOE2* and/or *APOE4* carrier status for each participant group.

For the purposes of the present analysis, the clinical syndromes were conventionally stratified into FTD (which has particularly marked clinicopathological heterogeneity), including bvFTD, svPPA and nfvPPA; PSP (a 4-repeat [4R] tauopathy); CBS (like FTD, relatively poorly predictive of a specific underlying proteinopathy); and FTD-MND (primarily related to a TDP-43 proteinopathy). All phenotypic variants were separately compared with HC and/or individuals with AD, with respect to *APOE2* and *APOE4* carriage.

Several subgroup analyses were specified a priori. bvFTD, svPPA and nfvPPA were each analysed separately compared with HC and AD groups, in terms of *APOE2* and *APOE4* carriage. Exploratory, pathology-restricted analyses were conducted selectively in subgroups where they were necessary to clarify the biological interpretation of *APOE* associations. In corticobasal syndromes, such analyses were performed due to the well-recognized overlap with AD pathology, which may confound APOE-related effects. In PSP, restriction to pathologically confirmed cases was undertaken in light of the unusual and biologically interesting observed *APOE2* and *APOE4* associations, which required further validation.

### 2.5. Statistical Analysis

RevMan 8.13.0 statistical software was utilized for all statistical analyses (https://revman.cochrane.org/info, last accessed on 30 April 2026). The level of statistical significance was conventionally set to *p* ≤ 0.003, to account for multiple comparisons (as the primary analysis was intended to include 16 meta-analyses). Results with *p*-values between 0.003 and 0.05 were considered nominal and directional associations and were not interpreted as statistically significant according to the prespecified multiplicity-adjusted threshold. Odds ratios (ORs) and their precision (95% confidence intervals [95% CIs]) were estimated for *APOE2* and *APOE4* carriage using as weights the inverse variance of individual effects. Heterogeneity among studies was statistically evaluated according to the Q and I^2^ statistics. Statistical heterogeneity was confirmed using Cochran’s Q test (P_Q_ < 0.1), and its magnitude was quantified using the I^2^ statistic (I^2^ between 30% and 50% was consistent with moderate and I^2^ > 50% was consistent with considerable heterogeneity). Given the anticipated clinical, methodological, and population heterogeneity among observational genetic association studies, random-effects models were used for all meta-analyses irrespective of the magnitude of statistical heterogeneity. ORs and 95% CIs were graphically depicted with forest plots.

Publication bias was assessed with funnel plots when 10 or more studies were synthesized. When statistical evidence of heterogeneity was observed in analyses including 10 or more studies, Baujat plots were utilized for the visualization of individual study contributions to the overall heterogeneity. Sensitivity analyses based on Baujat plots were conducted in order to confirm that our findings were not driven by the undue effects of individual studies; studies showing the greatest contribution to heterogeneity and/or influence on the pooled estimate were eliminated in the context of sensitivity analyses.

To assess the robustness of the findings, sensitivity analyses were conducted according to study quality. Main analyses were repeated after excluding studies with lower methodological quality (NOS score < 5). This approach aimed to minimize the potential influence of bias arising from study design and to determine whether the overall results were driven by lower-quality evidence. Consistency between the primary and restricted analyses was interpreted as supporting the stability and reliability of the observed associations.

As part of the revision process, additional post hoc exploratory analyses were performed to assess the potential influence of population stratification on the principal FTD associations. Studies were grouped as having a European/White population background when participants were explicitly described as European, White, Caucasian, or as belonging to a defined European population, and as having an East Asian population background when participants were explicitly described as Chinese or Japanese. Population background was not inferred solely from the country of recruitment, and studies with unreported, mixed, or other population backgrounds were excluded from these analyses. The principal comparisons of *APOE2* and *APOE4* carriage in FTD versus HC and AD were repeated within these groups when sufficient data were available.

## 3. Results

### 3.1. Study Characteristics

**Figure 1:**
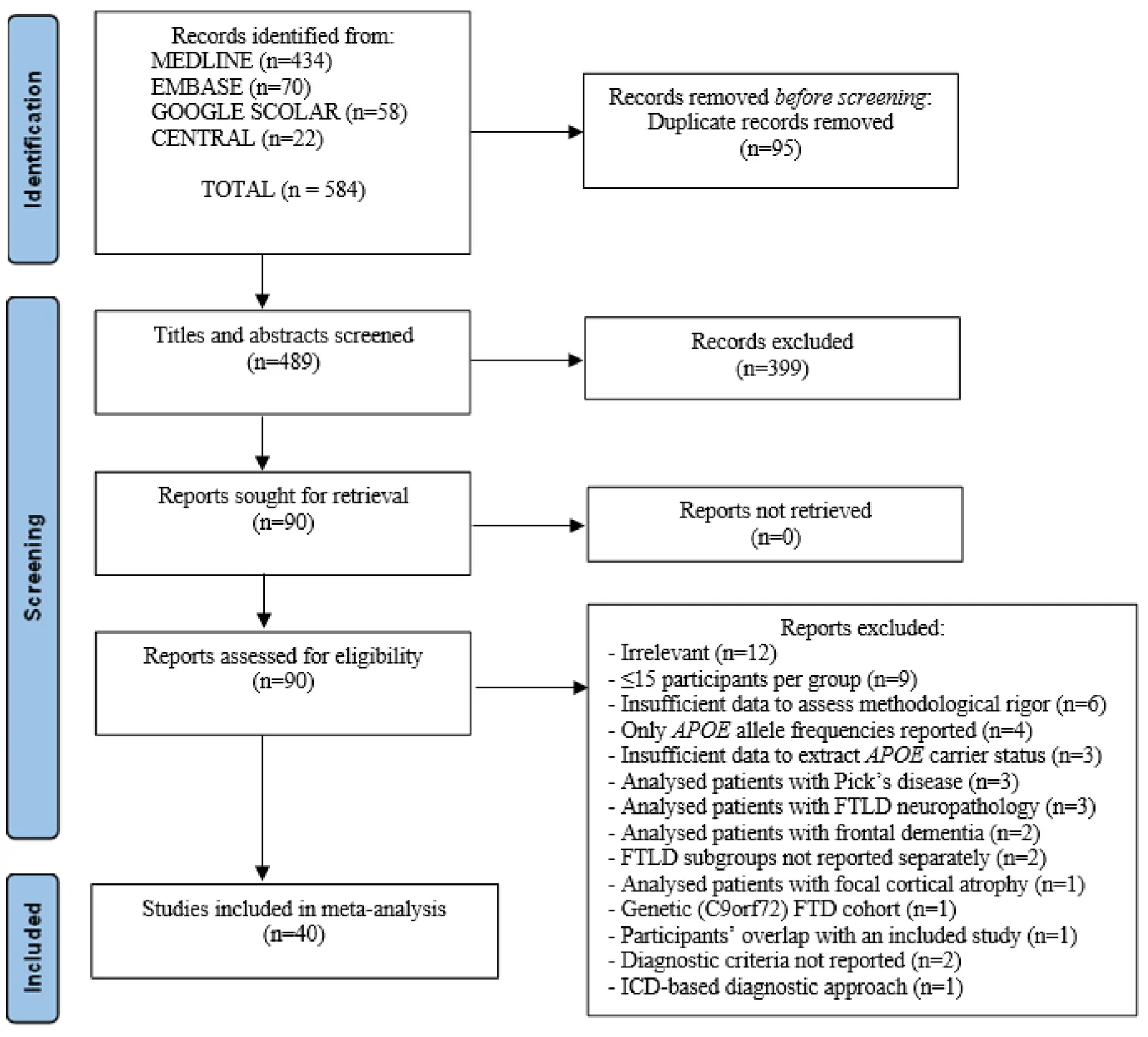
Flow diagram of study selection. *APOE*, apolipoprotein E; FTLD, frontotemporal lobar degeneration; *C9orf72*, chromosome 9 open reading frame 72; ICD, International Classification of Diseases; n, number.

The structured literature review identified 584 studies: 434 from MEDLINE (via PubMed), 70 from Embase (via Elsevier), 58 from Google Scholar, and 22 from CENTRAL. Following initial screening of titles and abstracts, 90 full-text articles were assessed for eligibility. Of these, 40 studies were included in the systematic review and meta-analysis (Figure 1).

Among the included studies, 31 involved participants with FTD [20,21,22,23,24,25,26,27,28,29,30,31,32,33,34,35,36,37,38,39,40,41,42,43,44,45,46,47,48,49,50], 8 with PSP [37,51,52,53,54,55,56,57], 5 with CBS or pathologically confirmed corticobasal degeneration (CBD) [37,52,54,55,58], and 3 with FTD-MND [24,41,59], with some studies including more than one phenotype. Detailed information on study settings, diagnostic approaches, and participant characteristics, including demographics, disease duration, and severity, are presented in Table S2.

### 3.2. FTD

**Figure 2:**
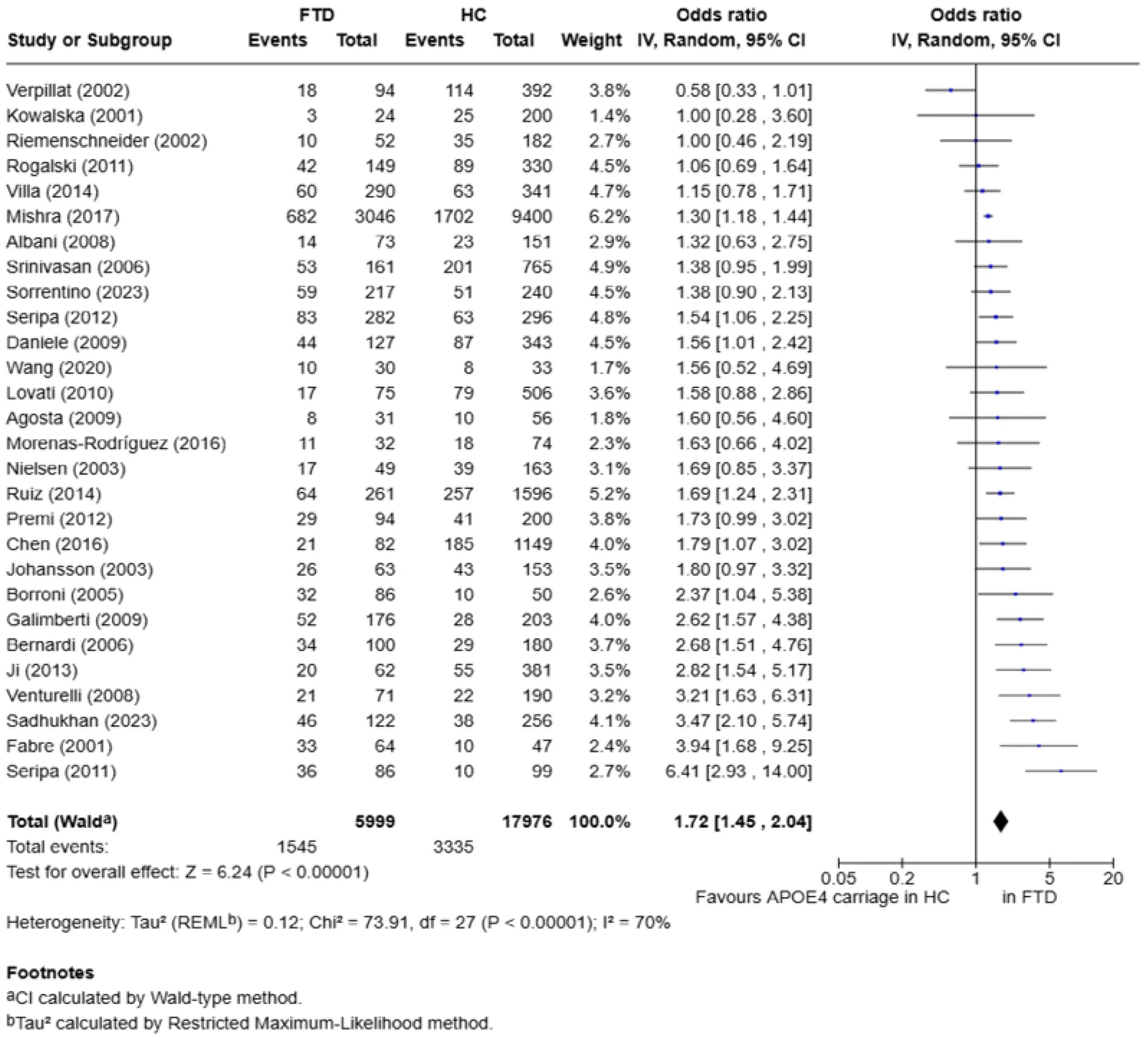
Meta-analyses of *APOE4* carriage status in frontotemporal dementia (FTD) vs. healthy controls (HCs). Studies included: Verpillat (2002) [48], Kowalska (2001) [50], Riemenschneider (2002) [47], Rogalski (2011) [33], Villa (2014) [28], Mishra (2017) [24], Albani (2008) [38], Srinivasan (2006) [41], Sorrentino (2023) [22], Seripa (2012) [31], Daniele (2009) [35], Wang (2020) [23], Lovati (2010) [34], Agosta (2009) [36], Morenas-Rodríguez (2016) [26], Nielsen (2003) [45], Ruiz (2014) [27], Premi (2012) [30], Chen (2016) [25], Johansson (2003) [46], Borroni (2005) [43], Galimberti (2009) [37], Bernardi (2006) [42], Ji (2013) [29], Venturelli (2008) [40], Sadhukhan (2023) [21], Fabre (2001) [49], and Seripa (2011) [32].

For the comparison of *APOE4* carriage between FTD and healthy controls (HCs), data from 28 studies were included, comprising 5999 individuals with FTD and 17,976 HCs. The pooled analysis showed that *APOE4* carriage was more frequent in the FTD group, indicating approximately a 72% higher likelihood of *APOE4* carriage compared with HC [OR = 1.72, 95% CI = (1.45–2.04), *p* < 0.001] (Figure 2). Due to the high heterogeneity (I^2^ = 70%), we performed a sensitivity analysis by excluding studies identified as highly influential/major contributors to heterogeneity based on Baujat plot inspection (Figure S1). After the removal of 4 studies, the pooled estimate became more precise [OR = 1.66, 95% CI = (1.46–1.89), *p* < 0.001] (Figure S2). The same 4 studies appeared outside the expected funnel limits in the funnel plot (Figure S3). The funnel plot showed no definitive asymmetry, but there was a slight tendency toward larger effects in smaller studies, which could reflect heterogeneity or small-study effects rather than clear publication bias.

**Figure 3:**
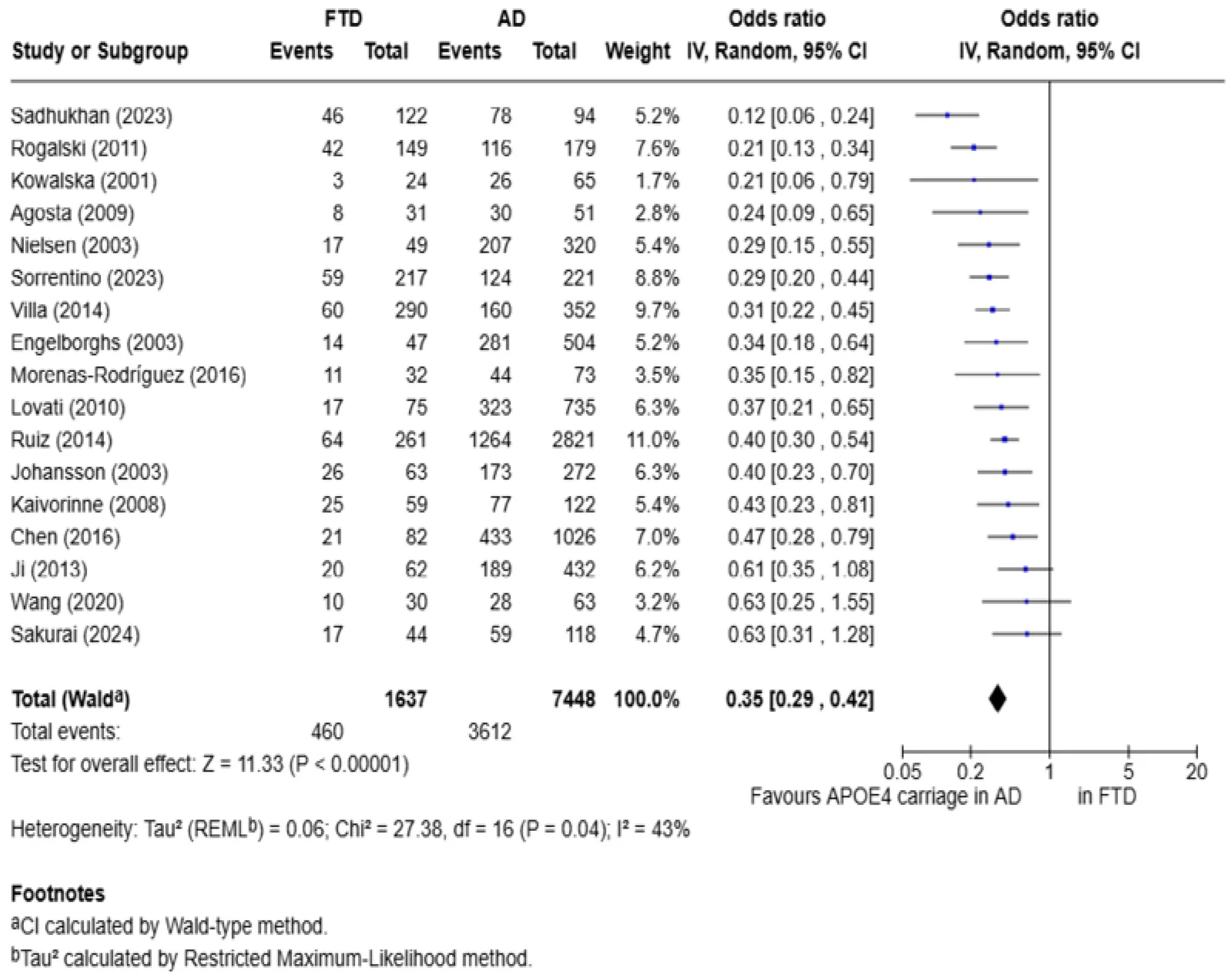
Meta-analyses of *APOE4* carriage status in frontotemporal dementia (FTD) vs. Alzheimer’s disease (AD). Studies included: Sadhukhan (2023) [21], Rogalski (2011) [33], Kowalska (2001) [50], Agosta (2009) [36], Nielsen (2003) [45], Sorrentino (2023) [22], Villa (2014) [28], Engelborghs (2003) [44], Morenas-Rodríguez (2016) [26], Lovati (2010) [34], Ruiz (2014) [27], Johansson (2003) [46], Kaivorinne (2008) [39], Chen (2016) [25], Ji (2013) [29], Wang (2020) [23], and Sakurai (2024) [20].

In the comparison between FTD and AD, 17 studies were pooled, including 1637 individuals with FTD and 7448 with AD. *APOE4* carriage was significantly less frequent in the FTD group, with 65% lower odds compared with AD [OR = 0.35, 95% CI = (0.29–0.42), *p* < 0.001] (Figure 3). Despite statistically significant heterogeneity (P_Q_ < 0.1), its magnitude was moderate (I^2^ = 43%), and the pooled estimates remained stable after removing the 3 highly influential studies/major contributors to heterogeneity (Figures S4 and S5). The same 3 studies fell outside or on the expected funnel boundaries in the funnel plot (Figure S6).

**Figure 4:**
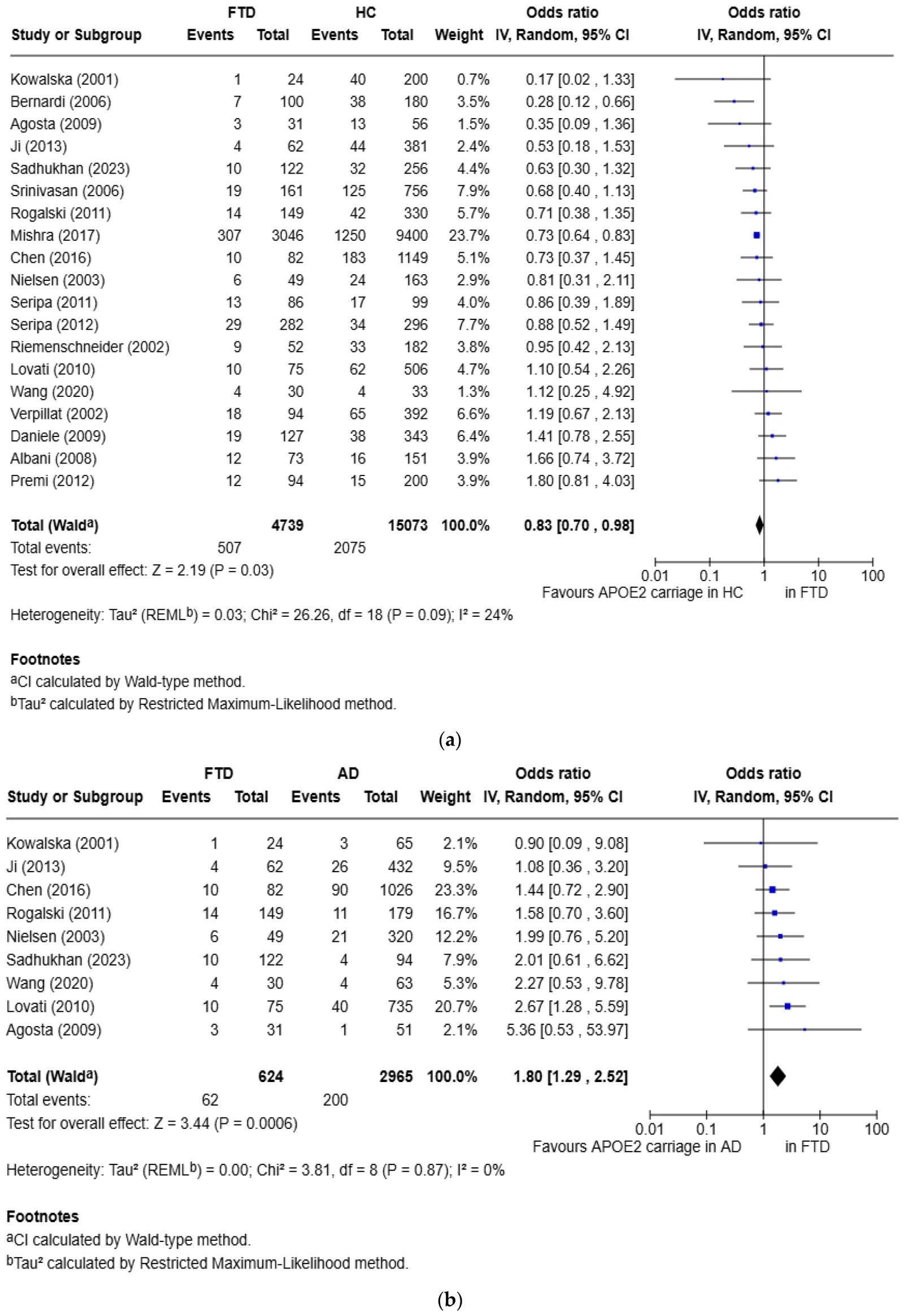
Meta-analyses of APOE2 carriage status (**a**) in frontotemporal dementia (FTD) vs. healthy controls (HCs) and (**b**) in FTD vs. Alzheimer’s disease (AD). Studies included: Kowalska (2001) [50], Bernardi (2006) [42], Agosta (2009) [36], Ji (2013) [29], Sadhukhan (2023) [21], Srinivasan (2006) [41], Rogalski (2011) [33], Mishra (2017) [24], Chen (2016) [25], Nielsen (2003) [45], Seripa (2011) [32], Seripa (2012) [31], Riemenschneider (2002) [47], Lovati (2010) [34], Wang (2020) [23], Verpillat (2002) [48], Daniele (2009) [35], Albani (2008) [38], and Premi (2012) [30].

Regarding *APOE2*, a quantitative synthesis of 19 studies including 4739 individuals with FTD and 15,073 HCs showed that the likelihood of *APOE2* carriage was nominally less frequent in the FTD group [OR = 0.83, 95% CI = (0.70–0.98), *p* = 0.03] (Figure 4a). Results were modestly heterogeneous (I^2^ = 24%)—though borderline statistically significant (*p* = 0.09)—and therefore sensitivity analysis based on Baujat plot inspection was performed (Figure S7). After removing 3 highly influential studies/major contributors to heterogeneity, the association was strengthened and met the adjusted significance threshold [OR = 0.76, 95% CI = (0.68–0.85), *p* < 0.001), while heterogeneity decreased to I^2^ = 0% (Figure S8). No evident asymmetry was observed in the funnel plot (Figure S9).

A pooled analysis of 9 studies comprising 624 participants with FTD and 2965 with AD revealed that *APOE2* carriage was more common in FTD, with approximately 80% higher odds than in AD [OR = 1.80, 95% CI = (1.29–2.52), *p* < 0.001] (Figure 4b). Findings were consistent across studies (I^2^ = 0%).

### 3.3. PSP

**Figure 5:**
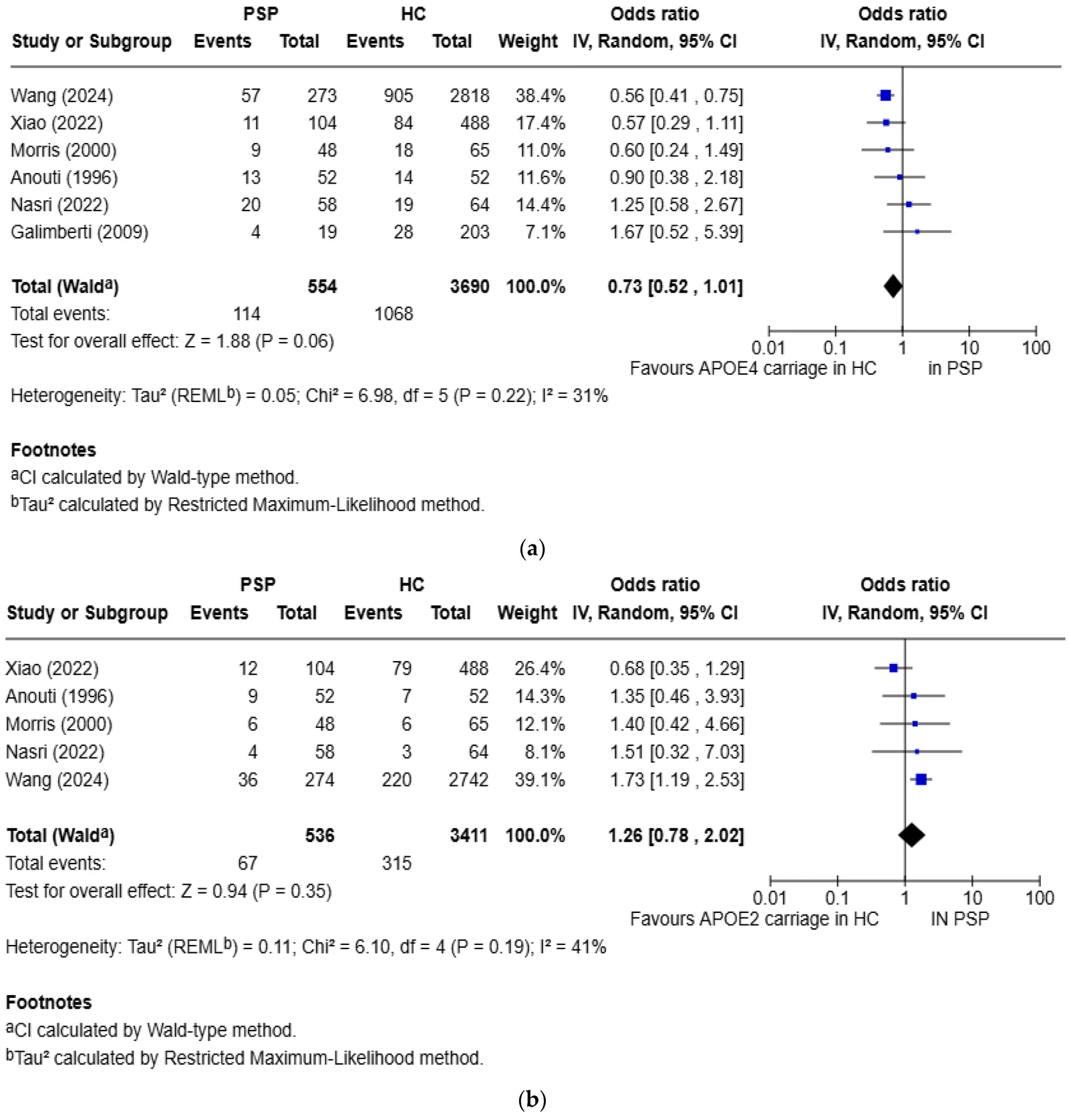
Meta-analyses of (**a**) *APOE4* carriage status in progressive supranuclear palsy (PSP) vs. healthy controls (HCs) and (**b**) *APOE2* carriage status in PSP vs. HC. Studies included: Wang (2024) [51], Xiao (2022) [53], Morris (2000) [56], Anouti (1996) [57], Nasri (2022) [52], and Galimberti (2009) [37].

Six studies encompassing 554 PSP cases and 3690 HCs compared *APOE4* carriage between PSP and HCs. The pooled results did not provide sufficient evidence of an association between *APOE4* carriage and PSP, although the point estimate indicated lower *APOE4* carriage in PSP than in HCs (Figure 5a). Five studies encompassing 536 PSP cases and 3411 HCs compared *APOE2* carriage between groups. The pooled analysis did not provide sufficient evidence of an association between *APOE2* carriage and PSP, although the point estimate indicated numerically higher *APOE2* carriage in PSP (Figure 5b).

**Figure 6:**
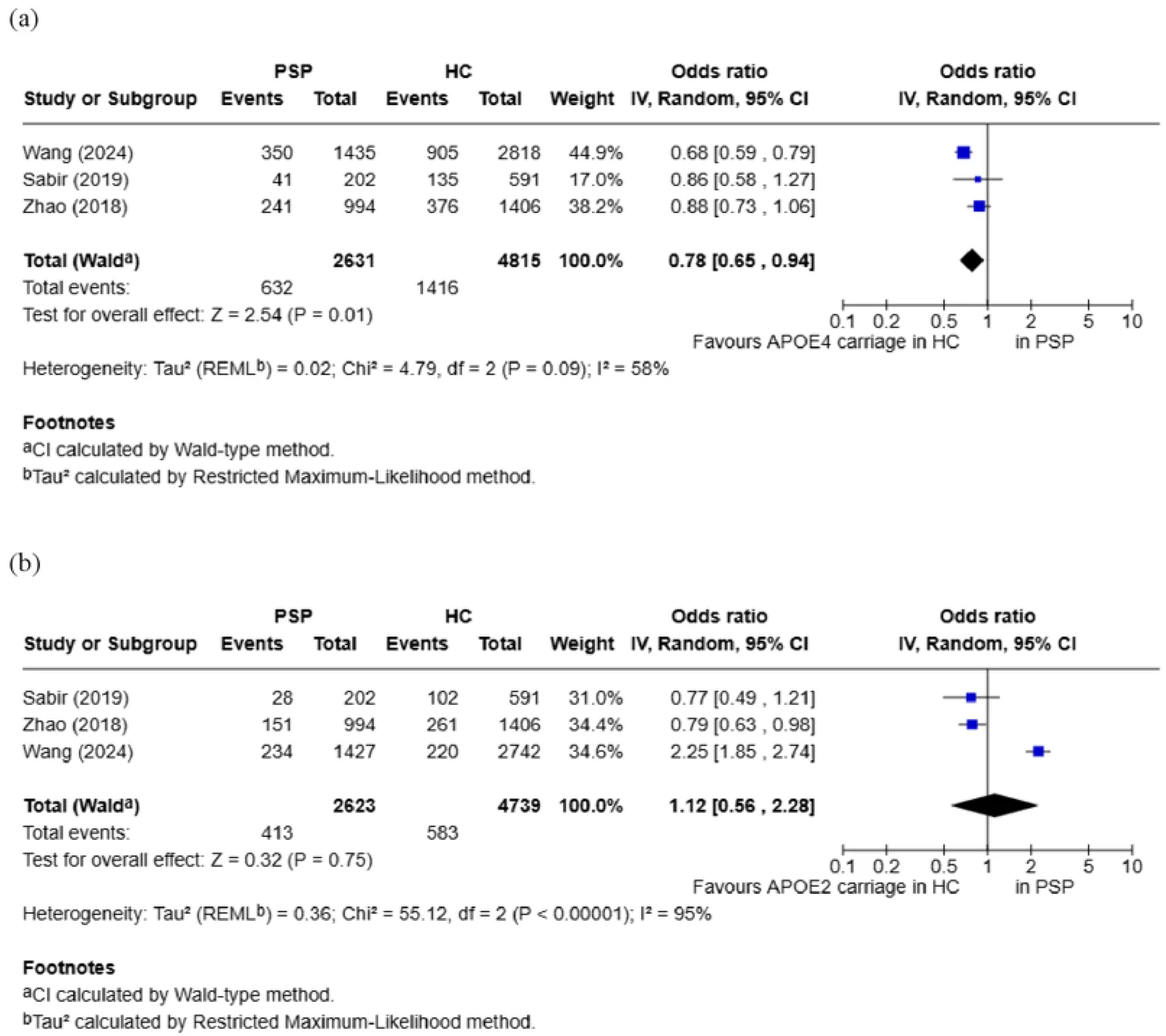
Exploratory meta-analyses of (**a**) *APOE4* carriage status in pathologically confirmed progressive supranuclear palsy (PSP) vs. healthy controls (HCs) and (**b**) *APOE2* carriage status in pathologically confirmed PSP vs. HC. Studies included: Wang (2024) [51], Sabir (2019) [54], and Zhao (2018) [55].

To further elucidate these biologically interesting findings, we performed an exploratory analysis including pathologically confirmed PSP cases. In these comparatively better powered analyses, *APOE4* carriage remained lower in PSP, showing a directionally consistent and nominally significant effect. Although strengthened, the association did not meet the prespecified multiplicity-adjusted threshold for statistical significance and was therefore considered exploratory rather than definitive (Figure 6a). In contrast, *APOE2* carriage was not associated with PSP in pathologically confirmed cohorts. Notably, existing evidence regarding *APOE2* in PSP was very inconsistent, with previous studies reporting directionally opposing associations (Figure 6b).

Finally, preliminary evidence from one study involving pathologically confirmed cases with PSP and AD supported pronounced differences in *APOE* genotype distribution between the two groups, with markedly lower *APOE4* [202 with PSP and 571 AD, OR = 0.18, 95% CI = (0.12–0.26), *p* < 0.001] and nominally higher *APOE2* carriage status in PSP compared with AD [OR = 2.14, 95% CI = (1.28–3.57), *p* = 0.004] [54].

### 3.4. CBS

Available data did not provide sufficient evidence to establish an association between *APOE4* carriage and CBS. The comparison with HC was based on a limited number of studies and participants with similar *APOE4* carriage between groups (Figure S10). In a single study including 31 individuals with CBS and 27 with AD, *APOE4* carriage was lower in CBS than in AD (OR = 0.18, 95% CI = 0.06–0.58, *p* = 0.004); given the small sample size and absence of replication, this finding should be considered preliminary [58]. Another study did not reveal any differences in terms of *APOE2* carriage between CBS and HCs [37 with CBS and 64 HCs, OR = 2.46, 95% CI = (0.52–11.68), *p* = 0.26] [52]. Exploratory analyses restricted to pathologically confirmed CBD cases did not provide sufficient evidence to establish associations with either *APOE4* or *APOE2* carriage; however, these analyses were based on very small sample sizes and cannot exclude potentially meaningful associations with certainty (Figure S11).

### 3.5. FTD-MND

Neither *APOE4* nor *APOE2* carriage met the predefined threshold for statistical significance in comparisons between FTD-MND and HC (Figure S12). Again, sample sizes were very small, and potentially meaningful associations cannot be excluded with certainty.

### 3.6. Subgroup Analyses

To evaluate whether the effects of *APOE* on FTD vary by phenotype, we conducted subgroup analyses (Figures S13–S22). The overall pattern of *APOE* associations was not uniform across FTD phenotypes. For *APOE4*, increased carriage relative to HC was most prominent in bvFTD, while a weaker association was observed in svPPA. When compared with AD, *APOE4* carriage was consistently lower across bvFTD and svPPA subtypes. For *APOE2*, carriage was reduced in bvFTD and nominally lower in svPPA compared with HC. In contrast, comparisons with AD did not yield significant differences, although these findings were based on limited data.

### 3.7. Sensitivity Analyses Based on Study Quality

Figures S23–S28 present the results of sensitivity analyses excluding lower-quality studies (NOS < 5) (Table S3). The findings were practically identical to those of the main analyses.

### 3.8. Exploratory Analyses According to Population Background

Exploratory analyses according to reported population background generally supported the robustness of the principal FTD findings (Figures S29–S35). For *APOE4* carriage in FTD versus HC, restriction to 13 studies with a European/White population background yielded significantly higher *APOE4* carriage in FTD [OR = 1.64, 95% CI = (1.22–2.20), *p* = 0.001], although substantial between-study heterogeneity remained (I^2^ = 84%). Similarly, analysis of 4 East Asian studies showed higher *APOE4* carriage in FTD than in HC [OR = 1.98, 95% CI = (1.39–2.83), *p* < 0.001; I^2^ = 0%].

The association between lower *APOE4* carriage and FTD relative to AD was also preserved across population groups. Among 5 studies with a European/White population background, *APOE4* carriage was less common in FTD than in AD [OR = 0.37, 95% CI = (0.31–0.45), *p* < 0.001; I^2^ = 0%]. A similar association was observed across 4 East Asian studies [OR = 0.51, 95% CI = (0.36–0.72), *p* < 0.001; I^2^ = 0%].

For *APOE2* carriage, the analysis restricted to 8 studies with a European/White population background showed nominally lower carriage in FTD than in HC (*p* = 0.03), but this association did not meet the prespecified multiplicity-adjusted significance threshold. In 4 East Asian studies, the direction of effect was likewise toward lower *APOE2* carriage in FTD, although the association was not statistically significant (*p* = 0.10). Finally, among 4 East Asian studies comparing FTD with AD, *APOE2* carriage did not differ significantly between groups (*p* = 0.22). A corresponding European/White-background analysis could not be meaningfully performed because of insufficient consistently reported population-background data.

Overall, the principal *APOE4* associations were consistent across European/White and East Asian populations, whereas population-specific evidence for *APOE2* remained less conclusive.

## 4. Discussion

In this meta-analysis, *APOE* showed distinct associations across the FTLD spectrum. *APOE4* carriage was more frequent in FTD compared with HC and less common than in AD. In contrast, *APOE2* carriage was less prevalent in FTD relative to HC but more frequent compared with in AD. Post hoc analyses according to reported population background showed directionally consistent APOE4 associations in European/White and East Asian subsets, suggesting that the principal findings were unlikely to be explained solely by differences in population composition. Subgroup analyses indicated that *APOE4* effects were most pronounced in bvFTD, with weaker associations in svPPA. *APOE2* effects appeared more consistent across the bvFTD and svPPA phenotypic subgroups. In pathologically confirmed PSP cases, *APOE4* carriage was nominally less frequent than in HC, although this finding did not meet the multiplicity-adjusted significance threshold. On the other hand, *APOE2* carriage was similar in PSP and HC. Evidence regarding nfvPPA, CBS/CBD and FTD-MND remained insufficient to establish or exclude associations with *APOE2* or *APOE4*. These exploratory analyses were limited by the small number of available studies and participants, and potentially meaningful effects cannot therefore be excluded.

Given that *APOE4* is a well-established risk factor for cerebral amyloid-β (Aβ) deposition and *APOE2* is associated with reduced Aβ burden, it could be argued that the *APOE* associations observed in FTD may partly reflect the presence of co-existing Aβ pathology within clinically defined FTD cohorts. However, co-pathology is unlikely to fully account for the present findings. Studies incorporating genetically or neuropathologically confirmed FTD cases reported that *APOE4* carriage can influence disease expression independent of Aβ co-pathology [17,60,61]. Accumulating experimental and translational evidence also indicated that *APOE2* and *APOE4* exert broader (opposing) effects on neurodegeneration through mechanisms independent of Aβ pathology [62,63,64]. Further, the association of *APOE4* with other non-AD and non-FTD dementia entities suggests that it may confer a more general susceptibility to neurodegenerative processes [65]. The finding that bvFTD appears to drive the *APOE4* association within the FTD spectrum may reflect its closer link to widespread degeneration and its marked clinicopathological heterogeneity. This heterogeneity may increase susceptibility to genetic modifiers such as *APOE*, whose effects are unlikely to be restricted to a single molecular pathway [62,63,66]. In contrast, PPA variants are more tightly linked to specific pathological substrates—svPPA predominantly to TDP-43 and nfvPPA more commonly to tau pathology—which may limit the influence of broader susceptibility factors [67].

*APOE* may influence neurodegeneration through its fundamental role in CNS lipid trafficking. Astrocyte-derived APOE-containing lipoproteins transport cholesterol and other lipids required for neuronal membrane remodeling and synapse formation [68]. Disturbances in lipid handling could alter membrane homeostasis, synaptic maintenance, and the capacity of neurons to respond to degenerative injury. Consistent with this concept, *APOE4* has been associated with abnormal intracellular cholesterol localization in oligodendrocytes, impaired myelination, and broader disturbances in cholesterol homeostasis [69]. Additional mechanisms potentially linking APOE to neurodegeneration include effects on redox balance and metal homeostasis, which may influence oxidative and lipid-peroxidative stress, although their relevance to FTLD remains hypothetical [70,71].

The relationship between *APOE* and tau pathology appears particularly complex and may be both isoform- and disease-context-dependent. In P301S tau-transgenic mice, *APOE4* increased neuroinflammation, tau accumulation, cerebral atrophy, and neuronal loss, demonstrating effects on tau-mediated neurodegeneration independent of Aβ [62]. Microglia appear to mediate a substantial component of this effect [72]. Conversely, *APOE2* cannot be assumed to exert uniformly protective effects in primary tauopathies. Experimental expression of human tau in *APOE2*-targeted replacement mice resulted in increased hyperphosphorylated tau species and tau aggregation, while *APOE2* carriage was associated with greater tau pathology in neuropathologically confirmed human PSP cases [55]. Taken together, these observations indicate that APOE isoforms may modify tau pathology in a context-dependent manner. Consequently, the biological effects attributed to *APOE2* and *APOE4* in AD should not be directly extrapolated to primary tauopathies such as PSP and CBD.

The nominal signal toward lower *APOE4* carriage in pathologically confirmed PSP cases is particularly intriguing. To date, there are no studies directly investigating the underlying pathophysiological basis of this connection, and such an effect should be interpreted with caution. Therefore, this exploratory association may reflect mechanisms beyond direct disease modification. One potential explanation is the presence of competing risks, whereby *APOE4* carriers are more likely to develop AD or mixed dementia, reducing their representation within clinically defined PSP cohorts. Survival bias may also contribute, as *APOE4* carriage has been associated with increased mortality in older populations, potentially leading to underrepresentation of *APOE4* carriers in cross-sectional cohorts and thereby accentuating an apparent inverse association with PSP [73]. In addition, diagnostic misclassification may operate in the opposite direction, with *APOE4*-associated cases preferentially diagnosed as AD or mixed pathologies rather than isolated PSP, further biasing observed genotype distributions. Cohort composition (differences in recruitment setting, diagnostic ascertainment, and neuropathological characterization), population stratification (variations across ancestral populations) and control allele frequencies may represent additional potential sources of confounding. Accordingly, in the absence of prospective longitudinal data, the present findings cannot determine whether this exploratory association reflects a biological effect or selection-related mechanisms.

Pooled data did not reveal an association between *APOE2* and PSP, although the point estimate indicated numerically higher *APOE2* carriage in clinically established PSP. Of note, previous studies reported an association of *APOE2* with increased tau pathology in the brains of PSP cases [55]. Experimental models have also demonstrated increased hyperphosphorylated tau species, tau aggregation, and behavioral abnormalities in *APOE2/2* mice [55]. Emerging evidence suggests that *APOE2* may also be associated with an increased risk of Parkinson’s disease (PD) and may influence motor phenotypes in AD, where it has been linked to parkinsonian signs [1,74]. Taken together, these observations may suggest that *APOE2* effects extend to neural systems underlying motor function in ageing brains. In this context, *APOE2* may not primarily act as a susceptibility factor for PSP per se, but rather as a modifier of disease expression. Specifically, APOE2 could influence the distribution and burden of tau pathology across neural networks, thereby shaping clinical phenotypes—particularly those involving motor systems. This framework may help explain the apparently conflicting findings in PSP pathological cohorts, where APOE2 does not consistently emerge as a risk allele but is nevertheless associated with increased tau pathology.

The evidence for nfvPPA, CBS/CBD and FTD-MND remains inconclusive because of the limited number and size of available studies. Accordingly, the observed effect estimates should be considered exploratory and require evaluation in larger, preferably pathology-characterized cohorts.

### Strengths and Limitations

This study has several important strengths. First, a meticulous methodological approach was adopted to account for heterogeneity, including separate analysis of studies based on phenotypic subtypes (clinical diagnoses) from those with neuropathologically confirmed cases, thereby improving interpretability. Second, the meta-analyses for FTD and PSP included large sample sizes, enhancing statistical power and yielding precise effect estimates. Third, extensive sensitivity analyses were performed to assess the robustness of the findings, demonstrating that the results remained consistent irrespective of study quality or the influence of studies contributing most to heterogeneity. Collectively, these methodological considerations strengthen the validity and reliability of the present findings.

However, despite these strengths, several limitations should be considered. First, the limited number of available studies for CBS, CBD, and FTD-MND reduces the precision of the estimated effects and may obscure potentially meaningful associations. Second, the absence of cohort studies limits the ability to explore temporal and causal relationships, which is particularly relevant for interpreting the nominal signal toward lower APOE4 carriage in pathologically confirmed PSP. Without longitudinal data, it remains unclear whether this observation reflects a true biological effect or is driven by competing risks, diagnostic selection, differential survival, or other sources of selection bias. Third, the overrepresentation of studies conducted in Western populations may limit the generalizability of these findings to other ethnic and geographic groups. Furthermore, variability in reporting across studies—allele-based analyses, lack of consistent distinction between FTLD subtypes, and so on—limited the extent to which available data could be fully capitalized upon in the present analyses.

Future studies should prioritize large, prospective, longitudinal designs with neuropathological confirmation to disentangle these effects. In addition, harmonization of diagnostic criteria and more detailed phenotypic characterization across studies would improve comparability and strengthen inference in this field. Finally, the integration of mechanistic approaches to elucidate the biological pathways underlying the observed associations is essential.

## 5. Conclusions

This systematic review and meta-analysis demonstrated that *APOE* associations vary across the FTLD spectrum. *APOE4* carriage was more frequent in FTD than in healthy controls but substantially less frequent than in AD, with the strongest signal observed in bvFTD. *APOE2* carriage was lower in FTD than in healthy controls and more frequent in FTD than in AD. In PSP, the available evidence did not establish an association with *APOE2* or *APOE4* in clinically defined cohorts, while pathology-restricted analyses showed a directionally consistent, nominal signal toward lower *APOE4* carriage that should be regarded as exploratory. Evidence for nfvPPA, CBS/CBD, and FTD-MND remains insufficient for firm conclusions.

## Data Availability

No new data were created or analyzed in this study. Data sharing is not applicable to this article.

## Supplementary Materials

The following supporting information can be downloaded at: https://www.mdpi.com/article/10.3390/ijms27177752/s1.

## Abbreviations

The following abbreviations are used in this manuscript:

APOE	apolipoprotein E
AD	Alzheimer’s disease
FTLD	frontotemporal lobar degeneration
FTD	frontotemporal dementia
bvFTD	behavioral variant frontotemporal dementia
svPPA	semantic variant primary progressive aphasia
nfvPPA	nonfluent/agrammatic variant primary progressive aphasia
CBS	corticobasal syndrome
PSP	progressive supranuclear palsy
FTD-MND	frontotemporal dementia with motor neuron disease
CBD	corticobasal degeneration
HC	healthy controls
NOS	Newcastle–Ottawa Scale
OR	odds ratio
CI	confidence interval
