# Supplementary material for: Apolipoprotein E Alleles Across the Spectrum of Frontotemporal Lobar Degeneration: A Systematic Review and Meta-Analysis
Source: Int J Mol Sci. 2026 Aug 29;27(17):7752. doi: 10.3390/ijms27177752 (PMC13565894; doi:10.3390/ijms27177752)
Supplement: Supplementary file 1 [file ijms-27-07752-s001.zip › ijms-4458379-supplementary.pdf]

Apolipoprotein E Alleles Across the Spectrum of Frontotemporal Lobar Degeneration: A Systematic Review and Meta-Analysis

Ioannis Liampas, Antonis Polyviou, Silvia Demiri, Gerasimos Malataras, Vasilis Oikonomakis, Vasileios Siokas, Odysseas Kargiotis, Zinovia-Maria Kefalopoulou, Elisabeth Chroni, Efthimios Dardiotis

Table S1 Quality assessment of case-control studies.

SELECTION	
1) Is the Case Definition Adequate?	Requires expert validation and use of internationally accepted clinical diagnostic criteria (+)
	Record linkage (e.g. ICD codes in database) or self-report with no reference to primary record or no description (-)
2) Representativeness of the Cases	All eligible cases with outcome of interest over a defined period of time, all cases in a defined catchment area, all cases in a defined hospital or clinic, group of hospitals, health maintenance organisation, or an appropriate sample of those cases (e.g. random sample) (+)
	Not satisfying requirements in part (a) or not stated (-)
3) Selection of Controls	For HC: community controls (i.e. same community as cases and would be cases if had outcome) (+)<br>Relatives or hospital controls, derived from a hospitalised population or no description (-)
	For AD: same hospital - clinic - group of hospitals (in general same origin) as cases (+)<br>Other hospital - clinic - group of hospitals (in general different origin), or no description (-)
4) Definition of Controls	For HC: HC without underlying neurodegenerative disorders (+)<br>Reporting underlying neurodegenerative disorders among HC (-)
	For AD: AD requires expert validation and use of internationally accepted clinical diagnostic criteria (+)<br>Record linkage (e.g. ICD codes in database) or self-report with no reference to primary record or no description (-)
COMPARABILITY	
Comparability of Cases and Controls on the Basis of the Design or Analysis (A maximum of 2 (HC) or 3 (AD) stars can be allotted in this category)	-HC must be matched for age and sex in the design of the study or analyses must be adjusted for age and sex.<br>-Individuals with AD must be matched for age, sex and an index of disease severity (either disease duration or cognitive performance, e.g., MMSE or MoCA)<br>-Statements of no differences between groups or that differences were not statistically significant (baseline comparisons) are not sufficient for establishing comparability.
EXPOSURE	
1) Ascertainment of Exposure	Established and validated molecular methods used for genotyping (+)
	Genotyping with non-validated molecular methods or methods not reported (-)
2) Call Rate	The call rate (% successful genotyping) similar in cases and controls (+)
	Call rate different in cases and controls or not reported (-)

Table S2 Study characteristics.

First name (year of publication)	Study design	Settings/ Country	Ancestry/ethnicity or population background	*APOE* genotyping method	Diagnostic approach and clinical/neuropathological confirmation	Participant groups	Age and sex	Disease duration – MMSE scores
Kurz (2025)	Designed as a prospective cohort study; analysed as a case-control study in the current meta-analysis	Patients were recruited from the outpatient clinics of the Departments of Psychiatry & Psychotherapy and Neurology at LMU Hospital, Munich, Germany.	Not reported	TaqMan SNP assay	Clinical diagnoses were established according to published clinical criteria (McKhann et al., 2011; Albert et al., 2011; Armstrong et al., 2013).<br><br>Diagnoses were supported by amyloid-based (CSF or PET) biomarkers.	AD (dementia or MCI): 26<br><br>CBS-Aβ (+): 8<br><br>CBS-Aβ (-): 26<br><br>HC: 17	AD: 17 females (65%), 70.1 years (6.9)<br><br>CBS-Aβ (+): 4 females (50%), 74.9 years (9.1)<br><br>CBS-Aβ (-): 15 females (58%), 71.2 years (6.1)<br><br>HC: 8 females (47%), 70.5 years (6.1)	**Duration**<br>AD: 37.95 months (23.89)<br><br>CBS-Aβ (+): 33.89 months (20.60)<br><br>CBS-Aβ (-): 30.12 months (17.39)<br><br>**MMSE**<br>AD: 25.0 (3.5)<br><br>CBS-Aβ (+): 19.6 (8.6)<br><br>CBS-Aβ (-): 24.5 (5.3)

Sakurai (2024)	Designed as a cross-sectional study; analysed as a case-control study in the current meta-analysis	Participants were recruited from multiple centres across Ontario, Canada, through the Ontario Neurodegenerative Disease Research Initiative (ONDRI).	Not fully reported; multiracial /ethnic composition	Standardized ONDRI protocol, validated by TaqMan allelic discrimination assay	Clinical diagnoses were established according to published clinical criteria (McKhann et al., 2011; Albert et al., 2011; Rascovsky et al., 2011; McKhann et al., 2011).	AD: 118<br><br>FTD: 44	Not reported separately for AD and FTD (demographics were stratified by *APOE4*/gait-speed groups, not diagnosis).	Not reported separately for AD and FTD (MMSE scores were stratified by *APOE4*/gait-speed groups, not diagnosis).
Wang (2024)	Case-control study	Participants with PSP were drawn from Alzheimer's Disease Sequencing Project (ADSP) whole-genome sequencing cohorts (PSP-NIH-CurePSP-Tau, PSP-CurePSP Tau, PSP-UCLA, and AMPAD-MAYO).<br><br>HC were drawn	Cases: European ancestry, genetically defined; HC self-identified as non-Hispanic White	Whole-genome sequencing at 30×coverage, with TaqMan assay/Sanger sequencing validation	Neuropathological diagnoses (1,441) were established according to published criteria (Hauw et al., 1994; Roemer et al., 2022)<br><br>Clinical diagnoses (277) were established according to published clinical criteria (NINDS-SPSP, 1996; MDS-PSP, 2017).	Autopsy confirmed PSP: 1,441<br><br>Clinically diagnosed PSP: 277<br><br>HC: 2,944	Autopsy confirmed PSP: 625 females (43%), 68.4 years (8.2)<br><br>Clinically diagnosed PSP: 129 females (46%), 65.7 years (7.7)<br><br>HC: 1,775 females (60%), 81.2 years (6.0)	Not reported

		from other cohorts in ADSP (includes international cohorts across multiple continents).						
Sadhukhan (2023)	Case-control study	Patients were recruited across Eastern India.<br><br>HC were recruited from Kolkata, India.	Ethnic Bengali population of Eastern India	PCR-RFLP with AflIII and HaeII digestion; Sanger sequencing	Clinical diagnoses were established according to published clinical criteria (McKhann et al., 2011; Rascovsky et al., 2011).	AD: 94<br><br>FTD: 114<br><br>HC: 256	AD: 42 females (44.7%), 58.3 years (9.8)*<br><br>FTD: 38 females (33.3%), 55.8 years (10.4)*<br><br>HC: 82 females (32.0%), age matched	**<u>MMSE:</u>**<br>AD: 14.0 (6.9)<br><br>FTD: 15.2 (8.7)<br><br>HC: ≥26

Sorrentino (2023)	Case-control study	Patients were recruited from the Alzheimer Unit of the Istituto di Ricovero e Cura a Carattere Scientifico (IRCCS) Fondazione Ca' Granda, Ospedale Maggiore Policlinico, University of Milan, Italy.	Not reported	TaqMan allelic discrimination on QuantStudio 12K Flex	Clinical diagnoses were established according to published clinical criteria (Dubois et al., 2014; Rascovsky et al., 2011).<br><br>Diagnoses were supported by MRI or CT. FDG-PET was performed in 225 cases.	AD: 221<br><br>FTD: 217<br><br>HC: 240	AD: 139 females (63%), 72.8 years (7.3)<br><br>FTD: 88 females (41%), 71.2 years (7.9)<br><br>HC: 166 females (69%), 77.3 years (6.8)	**MMSE:**<br>AD: 22.6 (4.9)<br><br>FTD: 23.1 (5.4)<br><br>HC: 29.1 (1.1)
Nasri (2022)	Case-control study	Patients were recruited from the department of Neurology at Razi University Hospital, Tunisia.	Tunisian	PCR-RFLP with HhaI digestion	Clinical diagnoses were established according to published clinical criteria (Höglinger et al., 2017; Armstrong et al., 2013).	PSP: 58<br><br>CBS: 37<br><br>HC: 64	PSP: 23 females (40%), 63.4 years (8.7)*<br><br>CBS: 16 females (43%), 64.5 (8.4)*<br><br>HC: 32 females (50%)	**MMSE:**<br>PSP: 17.2 (9.6)<br><br>CBS: 17.0 (9.1)
Xiao (2022)	Case-control study	Patients were recruited form the Department of Neurology at Xiangya	Chinese	Targeted gene sequencing (Illumina NovaSeq	Clinical diagnoses were established according to published clinical criteria (Hoglinger et al.,	PSP: 104<br><br>HC: 488	PSP: 43 females (41%), 64.9 years (7.4)	**Duration:**<br>PSP: 3.3 years (2.8)

		Hospital, Central South University, Hunan, China.<br><br>HC were recruited from the Physical Examination Center of the Xiangya Hospital.		6000)	2017).<br><br>Diagnoses were supported by MRI.		HC: 65.8 years (8.2)	
Wang (2020)	Case-control study	Patients were recruited from the cognitive impairment clinic of Tianjin, Huanhu Hospital, China.	Chinese	PCR followed by restriction enzyme digestion	Clinical diagnoses were established according to published clinical criteria (Rascovsky et al., 2011; McKhann et al., 2011).<br><br>Diagnoses in more than half of the patients were supported by PiB-PET and/or FDG-PET.	AD: 63<br><br>bvFTD: 30<br><br>HC: 33	AD: 33 females (52%), 66.3 years (9.6)<br><br>bvFTD: 22 females (73%), 64.5 years (7.7)<br><br>HC: 11 females (33%), 66.0 years (8.7)	**MMSE:**<br>AD: 16.2 (6.6)<br><br>bvFTD: 16.7 (8.7)<br><br>HC: 28.7 (1.2)
Sabir (2019)	Case-control study	Brain tissue and/or blood samples were obtained from eighteen North American and European research centers and	European ancestry (genetically defined)	Illumina NeuroChip array	Neuropathological diagnoses were established according to published criteria (Hyman et al., 2012; Hauw et al.,2019 & 1994; Dickson et al., 2002).	AD: 571<br><br>PSP: 202<br><br>CBD: 41<br><br>HC: 591	AD: 350 females (61%), 81.8 years (10.1)<br><br>CBD: 23 females (56%), 74.8 years (9.3)	Not reported

		brain banks.			HC were defined either pathologically (218) or clinically (373).		PSP: 89 females (44%), 78.1 years (9.7)<br><br>HC: 273 females (46%), 71.7 years (11.0)	
Zhao (2018)	Case-control study	Patients were drawn from the Mayo Clinic brain bank for neurodegenerative disorders, US.<br><br>HC were recruited as research participants at Mayo Clinic, US.	Non-Hispanic Caucasian	Custom TaqMan allelic discrimination assay (ABI 7900HT real-time PCR)	Neuropathological diagnoses were established according to published criteria (Hauw JJ et al., 1994; Dickson DW et al., 2002).	PSP: 994<br><br>CBD: 134<br><br>HC: 1406	PSP: 460 females (46%), 75 years (52, 98) †‡<br><br>CBD: 66 females (49%), 69 years (46, 96) †‡<br><br>HC: 782 females (56%), 75 years (45, 100) †‡	Not reported

Mishra (2017)	Case-control study	Participants were drawn from 44 international research groups (US; Canada; UK; France; Netherlands; Belgium; Germany; Denmark; Sweden; Spain; Italy; Australia).	European descent	Illumina SNP-array genotyping (Human 370K/550K/660K Quad and OmniExpress in discovery; NeuroX custom array in replication; Infinium platform), with 1000 Genomes-based genotype imputation using minimac; rs769449 used as proxy for rs429358 in the replication cohort	Clinical diagnoses were established according to published clinical criteria (Neary et al., 1998; Gorno-Tempini et al., 2011; Rascovsky et al., 2011).<br><br>Approximately 3% of cases were pathologically confirmed.	bvFTD: 2,067<br><br>nfvPPA: 490<br><br>svPPA: 497<br><br>FTD-MND: 294<br><br>HC: 9,400	Not reported<br><br>(cases and controls age- and ancestry-matched)	Not reported
Chen (2016)	Case-control study	Patients were recruited from memory disorders clinics in Huashan Hospital, China.	Chinese Han	TaqMan assay	Clinical diagnoses were established according to published clinical criteria (McKhann et al., 2011; McKhann et al., 2001).	AD: 1027<br><br>bvFTD: 28<br><br>svPPA: 54	AD: 575 females (60%), 68.1 years (9.5)*<br><br>bvFTD: 19 females (68%), 59.8	**MMSE**<br>AD: 14.4 (6.2)<br><br>bvFTD: 16.1 (8.1)<br><br>svPPA:

		HC were recruited from community epidemiologic investigations in China.				HC: 1,149	years (12.0)*<br><br>svPPA: 28 females (52%), 59.0 years (8.1)*<br><br>HC: 619 females (54%), 69.4 years (7.4)	14.6 (8.4)<br><br>HC: 28.5 (2.2)
Chiò (2016)	Case-control study	Patients were recruited from the Piemonte and Valle d'Aosta register for ALS, Italy.<br><br>HC were recruited through patients' general practitioners.	Italian	PCR followed by Sanger sequencing (BigDye; 3130 Genetic Analyzer)	Clinical diagnoses were established according to published clinical criteria (Brooks et al., 2000; Neary et al., 1998; Strong et al., 2009).	ALS: 357 (including 52 ALS-FTD)<br><br>HC: 223	ALS: 154 females (43%), 64.8 years (10.2)*<br><br>HC: age and sex matched	Not reported
Morenas-Rodríguez (2016)	Case-control study	Patients were recruited from the Memory Unit at Hospital Sant Pau, Spain.	Not reported	Previously described APOE genotyping method; assay not specified	Clinical diagnoses were established according to published clinical criteria (McKhann et al., 2011; Costa et al., 2013; Rascovsky et al., 2011; Gorno-Tempini et al., 2011; Litvan et al., 1996; Armstrong et	AD: 73<br><br>MCI (due to AD): 90<br><br>FTD: 32<br><br>PSP: 3	AD: 45 females (62%), 70 years (8.4)<br><br>MCI (due to AD): 49 females (54.4%), 67.9 years	**<u>MMSE:</u>**<br>AD: 27.4 (2.0)<br><br>FTD: 23.7 (7.2)<br><br>PSP + CBS: 23.1 (6.7)

					al., 2013).	CBS: 8<br><br>HC: 74	(8.7)<br><br>FTD: 11 females (34%), 64.6 years (9.1)<br><br>PSP + CBS: 8 females (73%), 71.2 years (NR)<br><br>HC: 51 females (69%), 59 years (8.3)	HC: 29.1 (1.0)
Ruiz (2014)	Case-control study	Participants were recruited from 11 specialised centers across Spain.	Spanish and of European origin	Not reported	Clinical diagnoses were established according to published clinical criteria (McKhann et al., 1984; Neary et al., 1998).	AD: 3,172 (*APOE* genotype available for 2,821)<br><br>FTD: 682 (*APOE* genotype available for 261)<br><br>HC: 2,169 (*APOE* genotype available	AD: 2,173 females (68.5%), 74.8 years (9.9)*<br><br>FTD: 319 females (46.8%), 64.8 years (9.9)*<br><br>HC: 1,262 females (58.2%), 75.4 years (10.3)	Not reported

						for 1,596)		
Villa (2014)	Case-control study	Patients were recruited from the Alzheimer Unit of Fondazione C` a Granda, IRCCS Ospedale Policlinico, Milan, Ospedale L. Sacco, Milan, Ospedale S. Raffaele Turro, Milan, IRCCS Istituto Centro San Giovanni di Dio Fatebenefratelli, Brescia, and University of Turin and IRCCS Multimedica, Milan, Italy.	Italian	PCR-RFLP (restriction fragment length polymorphism)	Clinical diagnoses were established according to published clinical criteria (NINCDS-ADRDA; Neary et al., 1998; McKhann et al., 2001).	AD: 352<br><br>FTD: 290<br><br>HC: 341	AD: 230 females (65%), 72.8 years (12.4)<br><br>FTD: 133 females (46%), 69.5 (11.8)<br><br>HC: 198 females (58%), 68.8 years (13.7)	**Duration:**<br>AD: 2.8 years (2.8)<br><br>FTD: 3.0 years (4.6)
Ji (2013)	Case-control study	Patients were recruited from Huanhu Hospital, Tianjin, China.	Chinese	PCR-RFLP — PCR amplification followed by CfoI digestion	Clinical diagnoses were established according to published clinical criteria (NINCDS-ADRDA; Neary et al., 1998).<br><br>Diagnoses were supported by MRI and	AD: 432<br><br>FTD: 62<br><br>HC: 381	AD: 244 females (57%), 72.0 years (8.6)<br><br>FTD: 36 females (58%), 68.8 years (6.9)	Not reported

					CT. PiB-PET and FDG-PET were performed in 29 cases (Cairns et al., 2007).		HC: 210 females (55%), 71.6 years (7.5)	
Premi (2012)	Case-control study	Patients were recruited from the Centre for Aging Brain and Neurodegenerative Disorders, University of Brescia, Italy, and from the Neuropsychology Unit at Department of Neurology, Rome University, Italy.	Not reported	PCR–RFLP, HhaI digestion	Clinical diagnoses were established according to published clinical criteria (Gorno-Tempini et al., 2011).	Total PPA: 94<br><br>nvfPPA: 66<br><br>svPPA: 28<br><br>HC: 200	PPA: 51 females (54%), 65.7 years (9.1)<br><br>HC: 98 females (49%), 63.8 years (7.6)	Not reported
Seripa (2012)	Case-control study	Patients were recruited from the Neuropsychology Unit of the Catholic University School of Medicine, Rome, and from the Centre for Aging Brain and Neurodegenera	Italian	TaqMan allelic discrimination	Clinical diagnoses were established according to published clinical criteria (Neary et al., 1998; McKhann et al., 2001; Mesulam 2003).<br><br>Diagnoses were supported by MRI.	Total FTD: 282<br><br>bvFTD: 207<br><br>PPA: 75<br><br>HC: 296	Total FTD: 144 females (51%), 67.5 (9.1)<br><br>bvFTD: 104 females (50.2%), 66.8 years (9.1)<br><br>PPA: 40 females (53.3%),	Not reported

		tive Disorders at the University of Brescia, Italy.<br><br>HC were recruited from previous studies in Italy.					69.7 years (8.9%)<br><br>HC: 130 females (43.9%), 66.3 (12.4)	
Seripa (2011)	Case-control study	Participants were recruited from the Neuropsychology Unit of the Department of Neurology at the Catholic University School of Medicine, Rome, Italy.	White; predominantly Italian	Not reported (It cites another paper, to which I do not have access)	Clinical diagnoses were established according to published clinical criteria (McKhann GM et al., 2001; Mesulam MM, 2001).	FTD: 86 (including 54 bvFTD and 32 PPA)<br><br>HC: 99	FTD: 50 females (58%), 69.4 years (8.4)<br><br>HC: 49 females (50%), 66.2 years (7.3)	Not reported
Rogalski (2011)	Case-control study	Participants were from the clinical core of the Northwestern Alzheimer's Disease Center (NADC), Chicago, US.	Not reported	PCR–RFLP restriction isotyping, HhaI digestion	Clinical diagnoses were established according to published clinical criteria (Mesulam MM., 2001 & 2003; McKhann GM et al., 1984).	Probable AD: 179<br><br>PPA: 149<br><br>HC: 330	Probable AD: 120 females (67%), 72.0 years (40 – 89) *†‡<br><br>PPA: 77 females (52%), 62.0 years (42-81)*†‡<br><br>HC: 249	Not reported

							females (76%), 70.0 years (50 – 91)†‡	
Lovati (2010)	Case-control study	Participants were recruited from the Alzheimer Units of Ospedale Maggiore Policlinico-IRCCS and Ospedale Sacco, Milan, and from the Presidio Ospedaliero di Magenta, Milan, Italy.	Not reported	PCR–RFLP, HhaI digestion	Clinical diagnoses were established according to published clinical criteria (NINCDS-ADRDA; Neary et al., 1998).	AD: 735<br><br>FTD: 75<br><br>HC: 506	AD: 529 females (72%), 73.8 years (9.8)*<br><br>FTD: 41 females (55%), 68.7 years (17.1)*<br><br>HC: 331 females (65%), 68.7 years (13.3)	Not reported
Daniele (2009)	Case-control study	Study based at the Institute of Neurology, Catholic University, Rome, Italy.	Not reported	Not reported	Clinical diagnoses were established according to published clinical criteria (Mesulam, 2001; McKhann et al., 2001)	FTD: 127<br><br>HC: 343	FTD: 67 females (53%), 67.3 years (9.7)<br><br>HC: 166 females (48%), 66.0 years (12.3)	Not reported
Agosta (2009)	Case-control study	Participants were recruited from Memory and Aging Center of University of	Not reported	PCR and Sanger sequencing	Clinical diagnoses were established according to published clinical criteria (NINCDS-ADRDA, Neary et al., 1998).	AD: 51 (21 *APOE4*-, 30 *APOE4*+)	AD, *APOE4*-: 14 females (67%), 67.9 years (11.2)	**MMSE:** AD, *APOE4*-: 21.6 (4.4)<br><br>AD,

		California, San Francisco, US.				bvFTD: 31 (23 *APOE4*-, 8 *APOE4*+)<br><br>HC: 56 (46 *APOE4*-, 10 *APOE4*+)	AD, *APOE4*+: 13 females (43%), 66.5 years (10.7)<br><br>bvFTD, *APOE4*-: 7 females (30%), 58.4 years (10.9)<br><br>bvFTD, *APOE4*+: 3 females (38%), 58.9 years (4.3)<br><br>HC, *APOE4*-: 27 females (59%), 66.5 years (9.4)<br><br>HC, *APOE4*+: 5 females (50%), 66.9 years (7.6)	*APOE4*+: 21.5 (6.2)<br><br>bvFTD, *APOE4*-: 22.7 (7.0)<br><br>bvFTD, *APOE4*+: 22.0 (7.8)<br><br>HC, *APOE4*-: 29.6 (0.6)<br><br>HC, *APOE4*+: 29.7 (0.7)
Galimberti (2009)	Case-control study	Patients were recruited from the Alzheimer Units of IRCCS, Ospedale Maggiore	Not reported	PCR–RFLP, HhaI digestion	Clinical diagnoses were established according to published clinical criteria (Neary et al., 1998; NINDS-SPSP; Litvan et al.,	bvFTD: 161<br><br>nfvPPA: 7	bvFTD: 91 females (57%), 65.4 years (10.2)*	<u>**Duration:**</u><br>bvFTD: 3.4 years (3.6)<br><br>nfvPPA:

		Policlinico, Ospedale Luigi Sacco, Ospedale S. Raffaele Turro, Milan, and IRCCS S. Giovanni di Dio-Fatebenefratelli, Brescia, Italy.			2003).<br><br>Diagnoses were supported with MRI or CT.	svPPA: 8<br><br>PSP: 19<br><br>CBS: 17<br><br>HC: 203	nfvPPA: 2 females (29%), 68.8 years (12.5)*<br><br>svPPA: 3 females (38%), 58.8 years (14.5)*<br><br>PSP:13 females (68%), 66.8 years (6.8)*<br><br>CBD/S: 11 females (65%), 66.4 years (9.4)*<br><br>HC: 113 females (56%), 64.3 years (10.8)	2.0 years (1.9)<br><br>svPPA: 3.8 years (5.1)<br><br>PSP: 3.2 years (2.7)<br><br>CBD/S: 2.1 years (0.5)
Albani (2008)	Case-control study	Participants were recruited from Fondazione Ospedale Maggiore Policlinico clinical center, Milan, and Luigi	Italian	PCR–RFLP, CfoI digestion	Clinical diagnoses were established according to published clinical criteria (Neary et al., 1998; McKhann et al., 2001).	FTD: 74<br><br>HC: 151	FTD: 40 females (57%), 69.4 years (9.7)*<br><br>HC: 90 females (60%), 67.0 years (12.0)	Not reported

		Sacco Hospital, Milan, Italy.						
Kaivorinne (2008)	Case-control study	Patients were recruited from the memory clinic of the Department of Neurology, Oulu University Hospital, Finland.<br><br>HC from blood donations at Finnish Red Cross offices in the capitals of the provinces of Northern and Central Ostrobothnia and Kainuu, Finland.	Finnish	PCR–RFLP, CfoI digestion	Clinical diagnoses were established according to published clinical criteria (Lund-Manchester; NINCDS-ADRDA).	Early-onset AD: 122<br><br>FTD: 59<br><br>HC: 198	Early-onset AD: 67 females (55%), 58.2 years, (38-64)†<br><br>FTD: 30 females (51%), 58.5 years (38-79)*†	Not reported
Venturelli (2008)	Case-control study	Participants were recruited from the Alzheimer Units of Ospedale Maggiore Policlinico IRCCS, Milan, Ospedale Sacco, Milan, and Ospedale San Raffaele	Not reported	PCR–RFLP, HhaI digestion	Clinical diagnoses were established according to published clinical criteria (Neary et al., 1998; McKhann et al., 2001; NINDS-SPSP; Litvan et al., 2003).<br><br>Diagnoses were supported with MRI or CT.	FTD: 71<br><br>PSP: 12<br><br>CBD: 6<br><br>HC: 190	FTD: 37 females (52%), 66.8 (8.3)*<br><br>PSP: 8 females (67%), 69.5 (4.5)*<br><br>CBD: 5 females	**Duration:**<br><br>FTD: 3.8 years (3.3)<br><br>PSP: 3.4 years (3.5)<br><br>CBD:2.2 years

		Turro, Milan, Italy.					(83%), 66.0 (7.0)*<br><br>HC: 106 females (56%), 65.7 years (11.0)	(0.4)
Srinivasan (2006)	Case-control study	Patients were recruited from the cerebral function unit of University of Manchester, England.<br><br>HC data were drawn from a cohort within the same Manchester region.	White	One-stage PCR	Clinical diagnoses were established according to published clinical criteria (Neary et al., 1998).<br><br>50 cases were pathologically confirmed (Brun et al., 1994).	bvFTD: 107<br><br>svPPA: 31<br><br>PPA (svPPA not included) : 23<br><br>FTD-MND: 30 progressive apraxia: 7<br><br>HC: 756	bvFTD: 53 females (49.5%), 57.4 years (9.2)*<br><br>svPPA: 19 females (61.3%), 60.6 years (7.1)*<br><br>PPA (svPPA not included): 9 females (39.1%), 65.7 years (6.4)*<br>FTD-MND: 11 females (36.7%), 61.6 years (8.4)*<br><br>HC: 529 females (70%), all	Not reported

							above 50 years	
Bernardi (2006)	Case control study	Patients were recruited from the Regional Neurogenetics Centre, Calabria, Italy.	Italian	PCR-RFLP using the Multigen APOE kit	Clinical diagnoses were established according to published clinical criteria (Lund-Manchester).	FTD: 100<br><br>HC: 180	FTD: 53 females (53%), 65.1 years (9.4)<br><br>HC: 96 females (53%), 65.2 years (6.7)	**Duration:**<br>FTD: 8.3 years (5.4)<br><br>**MMSE**:<br>FTD: 14.1 (7.2)<br><br>HC: 26 (3.7)
Borroni (2005)	Case-control study	Patients were recruited from the Early Onset Dementia Clinic at Addenbrookes Hospital, Cambridge, UK.<br><br>Controls were recruited from the same geographical area as the patients.	White, English	PCR–RFLP, HhaI digestion	Clinical diagnoses were established according to published clinical criteria (Neary et al. 1998).<br><br>13 cases were pathologically proven.	Total FTD: 86<br><br>bvFTD: 51<br><br>svPPA: 27<br><br>nfvPPA: 8<br><br>HC: 50	bvFTD: 33 females (65%), 55.5 years (7.8)*<br><br>svPPA: 7 females (26%), 59.0 years (6.9)*<br><br>nfvPPA: 3 females (38%), 58.3 years (8.4)*<br><br>HC: age and sex matched	Not reported
Engelborghs (2003)	Case-control study	Participants were recruited from the Department of	White, Belgian	PCR–RFLP, HhaI digestion	Clinical diagnoses were established according to published clinical criteria	AD: 504<br><br>FTD: 47	AD: 355 females (70%), 78.9 years (7.7)	**MMSE:**<br>AD: 14.2 (6.9)

		Neurology and Memory Clinic at Middelheim General Hospital, Antwerp, Belgium.			(NINCDS-ADRDA, 1984; DSM IV; Neary et al. 1998).	HC: 189	FTD: 19 females (40%), 69.0 years (10.3)<br><br>HC: 103 females (55%), 59.0 years (16.9)	FTD: 21.0 (6.3)
Nielsen (2003)	Case-control study	Participants were drawn from the Netherlands Brain Bank, Amsterdam, Netherlands.	Not reported	PCR-RFLP, CfoI digestion	Clinically established, neuropathologically confirmed diagnoses according to published criteria (Lund-Manchester; CERAD for AD).<br><br>All patients had been clinically assessed; dementia was established according to the current DSM-criteria.	AD: 320<br><br>FTD: 49<br><br>HC: 163	AD: 241 females (75%), 80.3 years (10.3)<br><br>FTD: 23 females (47%), 68.3 years (8.8)<br><br>HC: 72 females (44%), 73.5 years (13.2)	Not reported
Johansson (2003)	Case-control study	Not reported	Caucasian	Not reported	Clinical diagnoses were established according to published clinical criteria (Lund–Manchester; NINCDS-ADRDA).<br><br>93 AD cases and 103 HC were pathologically confirmed (AD	AD: 272<br><br>FTD: 70<br><br>HC: 160	AD: 167 females (61%), 75.1 years (9.9)<br><br>FTD: 42 females (60%), 63.8 years (8.0)	Not reported

					diagnosis was established according to CERAD).		HC: 90 females (56%), 73.9 years (9.3)	
Riemenschneider (2002)	Case-control study	Patients were recruited from a university memory clinic, Germany.<br><br>Most HC were spouses of patients from the memory clinic.	White, German	PCR-based restriction-enzyme genotyping	Clinical diagnoses were established according to published clinical criteria (Neary et al., 1998).<br><br>Diagnoses were supported by structural MRI, and functional imaging using either 99mTc HMPAO SPECT or FDG PET.	FTD: 52<br><br>HC: 182	FTD: 24 females (46%), 61.7 years (7.7)<br><br>HC: 97 females (53%), 63.9 years (9.3)	Not reported
Verpillat (2002)	Case-control study	Patients were recruited from 10 university hospitals in France.<br><br>HC were patient's spouses or healthy blood donors or individuals living in nursing homes.	White (living in France)	PCR–RFLP, HhaI digestion	Clinical diagnoses were established according to published clinical criteria (Lund-Manchester).	FTD: 94<br><br>HC:392	FTD: 55 females (58.5%), 60.6 years (8.5)*<br><br>HC:216 females (55.1%), 62.5 years (8.7)	Not reported
Fabre (2001)	Case-control study	Participants were recruited from the Geriatric Clinic of Huddinge	Sweden	Nonradioactive solid-phase microsequencing (AffiGene APOE)	Clinical diagnoses were established according to published clinical criteria (Lund–Manchester; Neary et	FTD: 65<br><br>HC: 51	FTD: 69% females, 59.4 years (9.1)*	Not reported

		University Hospital, Huddinge, and from the Sahlgrenska University Hospital, Mölndal, Sweden.			al., 1998).		HC: 70% females, 70.2 years (6.5)	
Kowalska (2001)	Case-control study	Partticipants were recruited from the Musashi Hospital, National Center of Neurology and Psychiatry, Tokyo.	Japanese	One-stage PCR	Clinical diagnoses were established according to published clinical criteria (Lund-Manchester; NINCDS-ADRDA; DSM-IV criteria)<br><br>Diagnoses were supported by CT, MRI, and SPECT.	AD: 501 (*APOE* genotype available for 65)<br><br>FTD: 24<br><br>HC: 200	Not reported	**<u>Duration:</u>** FTD: 4.0 years
Morris (2000)	Case-control study	Study based at UK academic centers.<br><br>HC were drawn from brain bank series.	White European	Not reported	Clinical diagnoses were established according to published clinical criteria (NINDS-SPSP).<br><br>HC and 13 PSP cases were pathologically confirmed.	PSP: 50<br><br>HC: 72	HC: 72 years	Not reported
Anouti (1996)	Case-control study	Patients with PSP were recruited from movement disorder clinics at the	Not reported	PCR–RFLP, HhaI digestion	Diagnoses were established clinically, prior to the first widely accepted clinical diagnostic criteria for PSP (bradykinesia,	PSP: 52<br><br>HC: 52	PSP: 20 females (38%), 71.0 years (6.4)<br><br>HC: 20	**<u>Duration:</u>** PSP: 3.6 years (3.3)

		University of Kansas, US, the Toronto Hospital, Buffalo Veterans Affairs Medical Center, and Robert Wood Johnson Medical School, Canada.<br><br>HC were recruited from movement disorder clinics at the University of Kansas, US.			rigidity with axial predominance, and supranuclear disorder of ocular motility with definite down-gaze palsy – progressive course, minimal and not sustained responsiveness to levodopa - patients with clinical features of multiple system atrophy or other causes of secondary parkinsonism were excluded).		females (38%), 70.9 years (6.8)	

Scale variables are presented as mean (SD); categorical variables are presented as absolute numbers (%); Age reported as age at recruitment or age at death; HC: healthy controls; FTD: frontotemporal dementia; FTLD: frontotemporal lobar degeneration; bvFTD: behavioral variant FTD; PPA: primary progressive aphasia; svPPA: semantic variant PPA; nfvPPA: nonfluent variant PPA; PSP: progressive supranuclear palsy; CBS/CBD: corticobasal syndrome/corticobasal degeneration; MCI: mild cognitive impairment; AD: Alzheimer's Disease; MND: motor neuron disease; MMSE: Mini-Mental State Examination; CT: computed tomography; MRI: magnetic resonance imaging; CSF: cerebrospinal fluid; PET: positron emission tomography; SPECT: single-photon emission computed tomography; PiB: Pittsburgh compound B; FDG: ^{18}F-fluorodeoxyglucose; * Age at onset; † Age variability reported as range (min–max); ‡ Age reported as median age.

Table S3 Quality analysis based on Newcastle-Ottawa Scale.

Case-control studies with healthy controls				
Study	Selection	Comparability	Exposure	NOS Score
Kurz (2025)	***	**	*	6
Wang (2024)	***	*	*	5
Sadhukhan (2023)	**	*	*	4
Sorrentino (2023)	**	*	*	4
Nasri (2022)	***	**	*	6
Xiao (2022)	***	**	*	6
Wang (2020)	*	**	*	4
Sabir (2019)	*	**	**	5
Zhao (2018)	**	**	*	5
Mishra (2017)	***	*	*	5
Chen (2016)	***	**	*	6
Chiò (2016)	***	**	*	6
Morenas-Rodríguez (2016)	***	*	*	5
Ruiz (2014)	**	**	*	5
Villa (2014)	***	*	*	5
Ji (2013)	****	**	*	7
Premi (2012)	**	*	*	4
Seripa (2012)	***		*	4
Seripa (2011)	***	**	*	6
Rogalski (2011)	**	**	*	5
Lovati (2010)	**	**	*	5
Daniele (2009)	*	*	*	3
Agosta (2009)	**	**	*	5
Galimberti (2009)	**	**	*	5
Albani (2008)	*	*	*	3
Venturelli (2008)	**	**	*	5
Srinivasan (2006)	***	**	*	6
Bernardi (2006)	***	**	*	6
Borroni (2005)	***	**	*	6
Nielsen (2003)	***		*	4
Johansson (2003)	**		*	3
Riemenschneider (2002)	**	**	*	5
Verpillat (2002)	**	**	*	5
Fabre (2001)	*		*	2
Kowalska (2001)	**		*	3
Morris (2000)	**		*	3
Anouti (1996)	**	**	*	5
Case-control studies with individuals with Alzheimer’s disease as controls				
Kurz (2025)	****	**	*	7
Sakurai (2024)	***		*	4
Sadhukhan (2023)	***		*	4

Sorrentino (2023)	****		*	5
Wang (2020)	***		*	4
Sabir (2019)	**		**	4
Chen (2016)	****		*	5
Morenas-Rodríguez (2016)	****	*	*	6
Ruiz (2014)	****		*	5
Villa (2014)	****		*	5
Ji (2013)	****		*	5
Rogalski (2011)	****	**	*	7
Lovati (2010)	****		*	5
Agosta (2009)	****		*	5
Kaivorinne (2008)	****		*	5
Engelborghs (2003)	***		*	4
Nielsen (2003)	**		*	3
Johansson (2003)	**		*	3
Kowalska (2001)	**		*	3

Figure S1 Baujat plot for the meta-analysis of *APOE4* carriage in frontotemporal dementia (FTD) vs. healthy controls (HC).

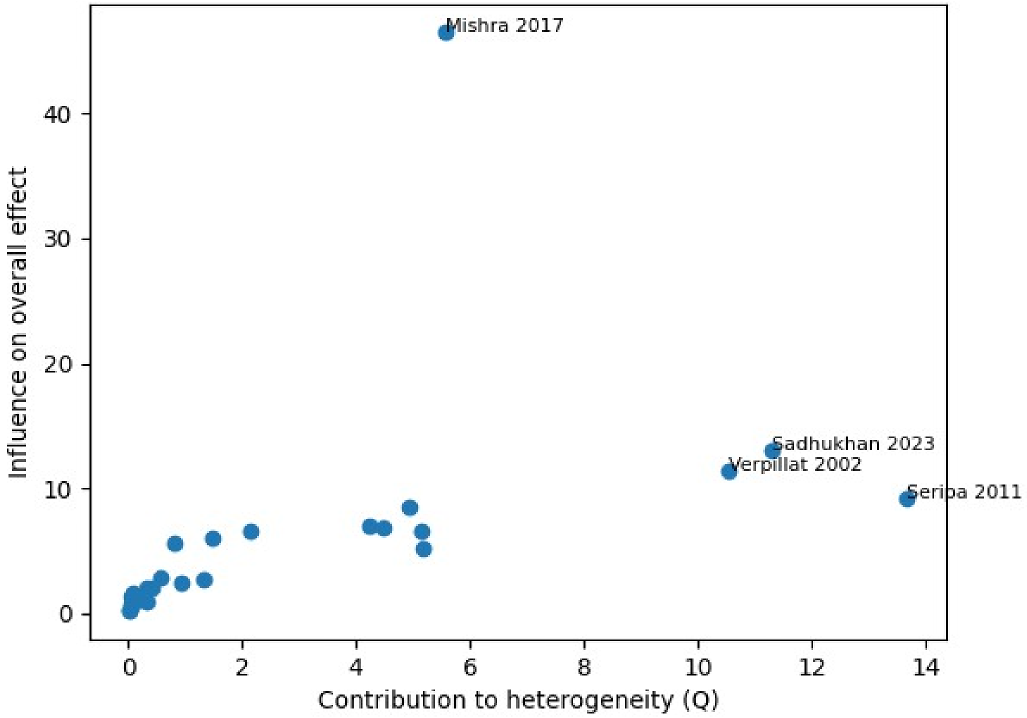

Figure S2 Sensitivity analysis of *APOE4* carriage in frontotemporal dementia (FTD) vs. healthy controls (HC), based on the Baujat plot.

Study or Subgroup	Events	Total	Events	Total	Weight	Odds ratio IV, Random, 95% CI
✗ Verpillat (2002)	18	94	114	392	0.0%	0.58 [0.33 , 1.01]
✓ Kowalska (2001)	3	24	25	200	1.0%	1.00 [0.28 , 3.60]
✓ Riemenschneider (2002)	10	52	35	182	2.4%	1.00 [0.46 , 2.19]
✓ Rogalski (2011)	42	149	89	330	6.2%	1.06 [0.69 , 1.64]
✓ Villa (2014)	60	290	63	341	7.0%	1.15 [0.78 , 1.71]
✗ Mishra (2017)	682	3046	1702	9400	0.0%	1.30 [1.18 , 1.44]
✓ Albani (2008)	14	73	23	151	2.7%	1.32 [0.63 , 2.75]
✓ Srinivasan (2006)	53	161	201	765	7.7%	1.38 [0.95 , 1.99]
✓ Sorrentino (2023)	59	217	51	240	6.2%	1.38 [0.90 , 2.13]
✓ Seripa (2012)	83	282	63	296	7.4%	1.54 [1.06 , 2.25]
✓ Daniele (2009)	44	127	87	343	6.1%	1.56 [1.01 , 2.42]
✓ Wang (2020)	10	30	8	33	1.3%	1.56 [0.52 , 4.69]
✓ Lovati (2010)	17	75	79	506	3.9%	1.58 [0.88 , 2.86]
✓ Agosta (2009)	8	31	10	56	1.4%	1.60 [0.56 , 4.60]
✓ Morenas-Rodríguez (2016)	11	32	18	74	1.9%	1.63 [0.66 , 4.02]
✓ Nielsen (2003)	17	49	39	163	3.0%	1.69 [0.85 , 3.37]
✓ Ruiz (2014)	64	261	257	1596	9.3%	1.69 [1.24 , 2.31]
✓ Premi (2012)	29	94	41	200	4.3%	1.73 [0.99 , 3.02]
✓ Chen (2016)	21	82	185	1149	4.7%	1.79 [1.07 , 3.02]
✓ Johansson (2003)	26	63	43	153	3.6%	1.80 [0.97 , 3.32]
✓ Borroni (2005)	32	86	10	50	2.2%	2.37 [1.04 , 5.38]
✓ Galimberti (2009)	52	176	28	203	4.8%	2.62 [1.57 , 4.38]
✓ Bernardi (2006)	34	100	29	180	4.1%	2.68 [1.51 , 4.76]
✓ Ji (2013)	20	62	55	381	3.7%	2.82 [1.54 , 5.17]
✓ Venturelli (2008)	21	71	22	190	3.1%	3.21 [1.63 , 6.31]
✗ Sadhukhan (2023)	46	122	38	256	0.0%	3.47 [2.10 , 5.74]
✓ Fabre (2001)	33	64	10	47	2.1%	3.94 [1.68 , 9.25]
✗ Seripa (2011)	36	86	10	99	0.0%	6.41 [2.93 , 14.00]
**Total (Wald[a])**		**2651**		**7829**	**100.0%**	**1.66 [1.46 , 1.89]**
Total events:	763		1471			
Test for overall effect: Z = 7.73 (P < 0.00001)						

Heterogeneity: Tau^2 (REML[b]) = 0.02; Chi^2 = 29.08, df = 23 (P = 0.18); I^2 = 22%

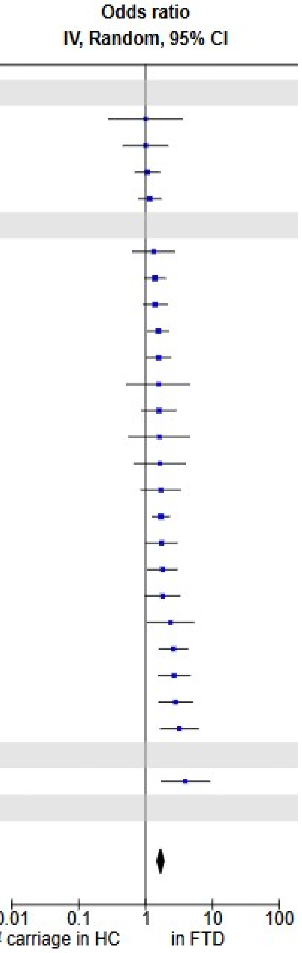

Footnotes

[a]CI calculated by Wald-type method.

[b]Tau^2 calculated by Restricted Maximum-Likelihood method.

Figure S3 Funnel plot for the meta-analysis of *APOE4* carriage in frontotemporal dementia (FTD) vs. healthy controls (HC).

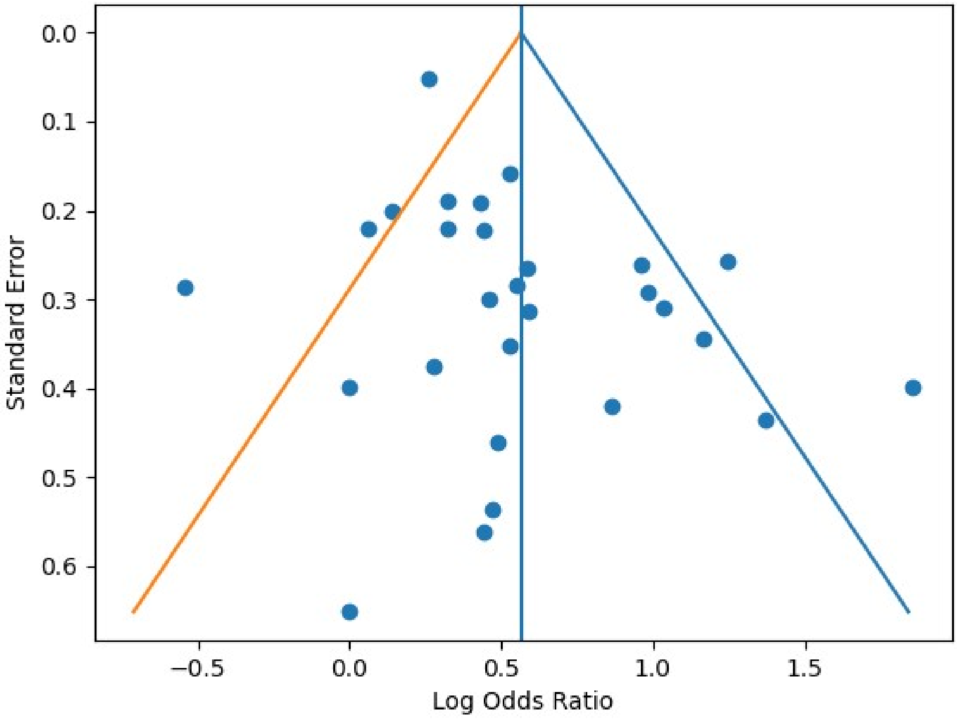

Figure S4 Baujat plot for the meta-analysis of *APOE4* carriage in frontotemporal dementia (FTD) vs. Alzheimer's disease (AD).

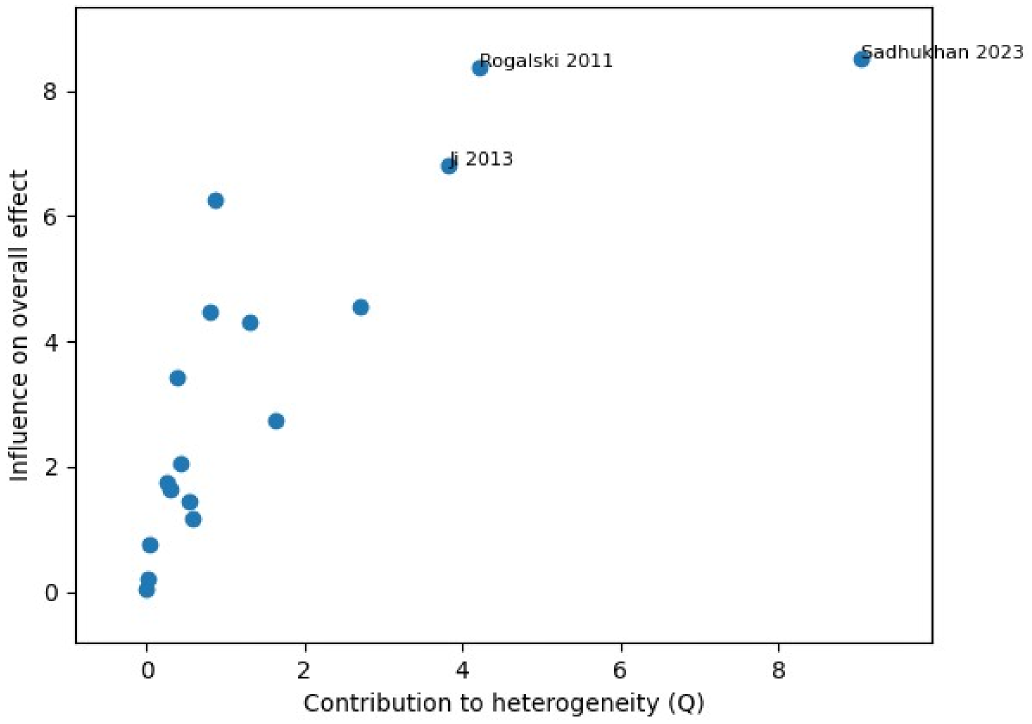

Figure S5 Sensitivity analysis of *APOE4* carriage in frontotemporal dementia (FTD) vs. Alzheimer's disease (AD), based on the Baujat plot.

Study or Subgroup	Events	Total	Events	Total	Weight	Odds ratio IV, Random, 95% CI
✕ Sadhukhan (2023)	46	122	78	94	0.0%	0.12 [0.06 , 0.24]
✕ Rogalski (2011)	42	149	116	179	0.0%	0.21 [0.13 , 0.34]
✓ Kowalska (2001)	3	24	26	65	1.2%	0.21 [0.06 , 0.79]
✓ Agosta (2009)	8	31	30	51	2.1%	0.24 [0.09 , 0.65]
✓ Nielsen (2003)	17	49	207	320	5.0%	0.29 [0.15 , 0.55]
✓ Sorrentino (2023)	59	217	124	221	12.6%	0.29 [0.20 , 0.44]
✓ Villa (2014)	60	290	160	352	16.1%	0.31 [0.22 , 0.45]
✓ Engelborghs (2003)	14	47	281	504	4.8%	0.34 [0.18 , 0.64]
✓ Morenas-Rodríguez (2016)	11	32	44	73	2.7%	0.35 [0.15 , 0.82]
✓ Lovati (2010)	17	75	323	735	6.4%	0.37 [0.21 , 0.65]
✓ Ruiz (2014)	64	261	1264	2821	23.6%	0.40 [0.30 , 0.54]
✓ Johansson (2003)	26	63	173	272	6.4%	0.40 [0.23 , 0.70]
✓ Kaivorinne (2008)	25	59	77	122	5.0%	0.43 [0.23 , 0.81]
✓ Chen (2016)	21	82	433	1026	7.7%	0.47 [0.28 , 0.79]
✕ Ji (2013)	20	62	189	432	0.0%	0.61 [0.35 , 1.08]
✓ Wang (2020)	10	30	28	63	2.4%	0.63 [0.25 , 1.55]
✓ Sakurai (2024)	17	44	59	118	4.0%	0.63 [0.31 , 1.28]
**Total (Wald[a])**		**1304**		**6743**	**100.0%**	**0.37 [0.32 , 0.42]**
Total events:	352		3229			
Test for overall effect: Z = 13.83 (P < 0.00001)						

Odds ratio
IV, Random, 95% CI

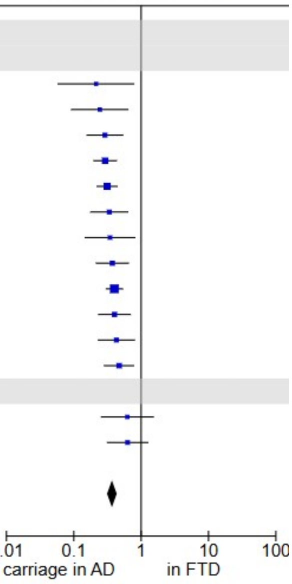

Heterogeneity: Tau² (REML[b]) = 0.00; Chi² = 9.15, df = 13 (P = 0.76); I² = 0%

Footnotes

[a]CI calculated by Wald-type method.

[b]Tau² calculated by Restricted Maximum-Likelihood method.

Figure S6 Funnel plot for the meta-analysis of *APOE4* carriage in frontotemporal dementia (FTD) vs. Alzheimer's disease (AD).

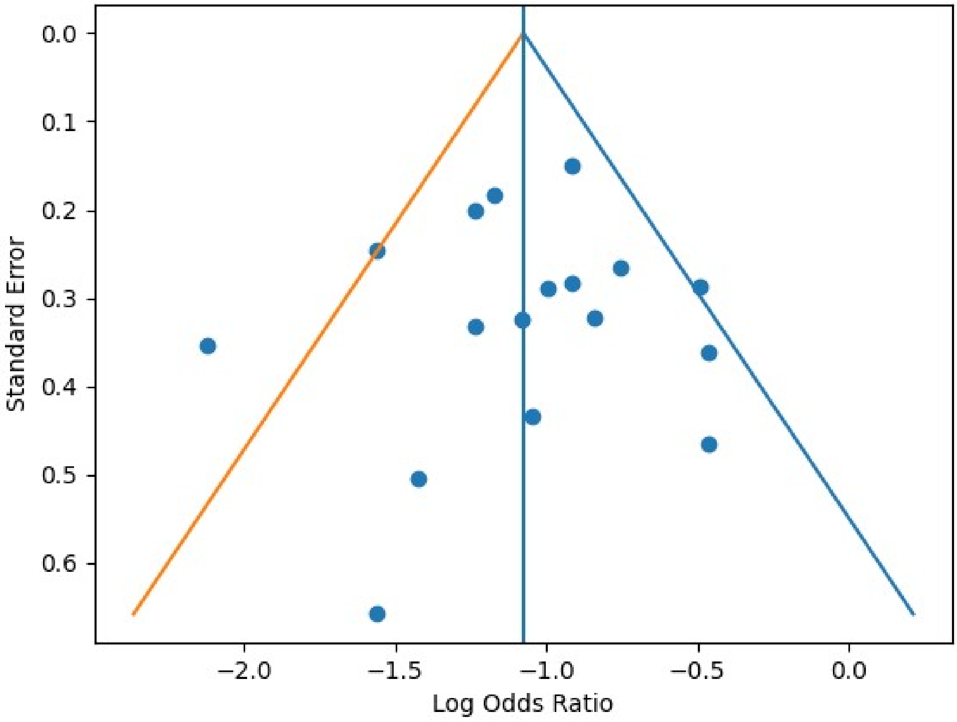

Figure S7 Baujat plot for the meta-analysis of *APOE2* carriage in frontotemporal dementia (FTD) vs. healthy controls (HC).

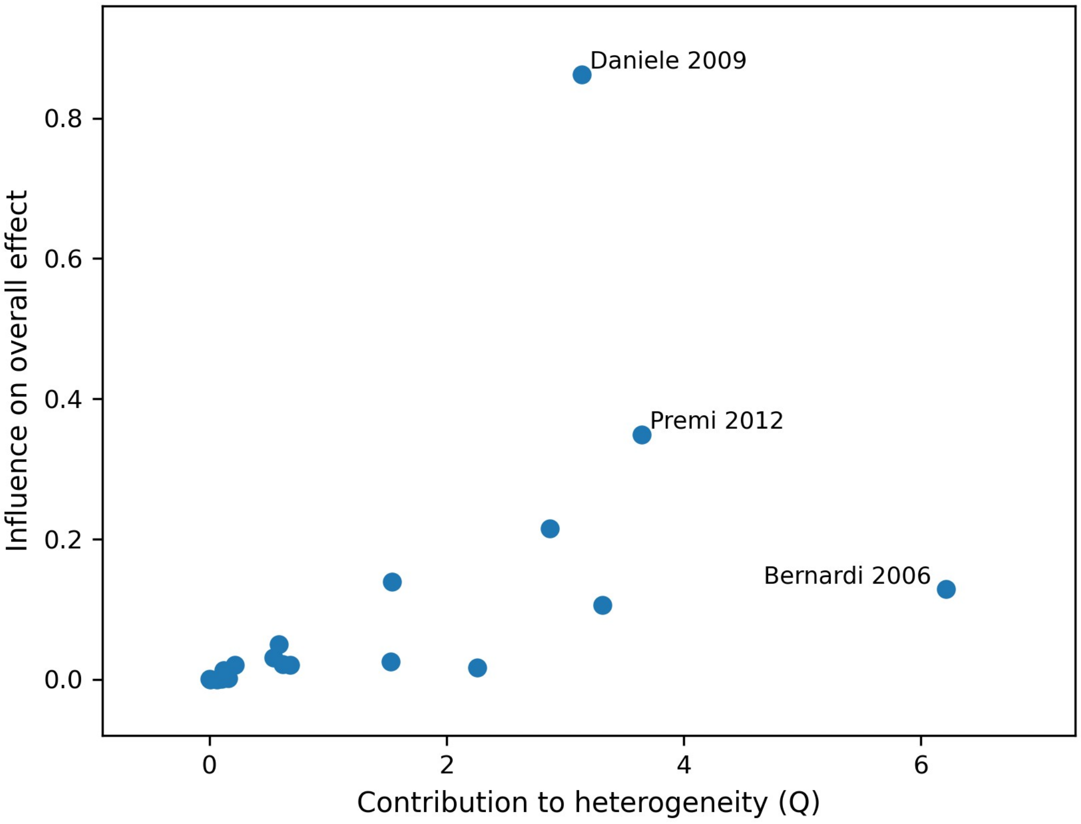

Figure S8 Sensitivity analysis of *APOE2* carriage in frontotemporal dementia (FTD) vs. healthy controls (HC), based on the Baujat plot.

Study or Subgroup	Events	Total	Events	Total	Weight	Odds ratio IV, Random, 95% CI
✓ Kowalska (2001)	1	24	40	200	0.3%	0.17 [0.02 , 1.33]
✕ Bernardi (2006)	7	100	38	180	0.0%	0.28 [0.12 , 0.66]
✓ Agosta (2009)	3	31	13	56	0.7%	0.35 [0.09 , 1.36]
✓ Ji (2013)	4	62	44	381	1.1%	0.53 [0.18 , 1.53]
✓ Sadhukhan (2023)	10	122	32	256	2.1%	0.63 [0.30 , 1.32]
✓ Srinivasan (2006)	19	161	125	756	4.5%	0.68 [0.40 , 1.13]
✓ Rogalski (2011)	14	149	42	330	2.9%	0.71 [0.38 , 1.35]
✓ Mishra (2017)	307	3046	1250	9400	68.3%	0.73 [0.64 , 0.83]
✓ Chen (2016)	10	82	183	1149	2.6%	0.73 [0.37 , 1.45]
✓ Nielsen (2003)	6	49	24	163	1.3%	0.81 [0.31 , 2.11]
✓ Seripa (2011)	13	86	17	99	1.9%	0.86 [0.39 , 1.89]
✓ Seripa (2012)	29	282	34	296	4.3%	0.88 [0.52 , 1.49]
✓ Riemenschneider (2002)	9	52	33	182	1.8%	0.95 [0.42 , 2.13]
✓ Lovati (2010)	10	75	62	506	2.3%	1.10 [0.54 , 2.26]
✓ Wang (2020)	4	30	4	33	0.5%	1.12 [0.25 , 4.92]
✓ Verpillat (2002)	18	94	65	392	3.6%	1.19 [0.67 , 2.13]
✕ Daniele (2009)	19	127	38	343	0.0%	1.41 [0.78 , 2.55]
✓ Albani (2008)	12	73	16	151	1.8%	1.66 [0.74 , 3.72]
✕ Premi (2012)	12	94	15	200	0.0%	1.80 [0.81 , 4.03]
**Total (Wald[a])**		**4418**		**14350**	**100.0%**	**0.76 [0.68 , 0.85]**
Total events:	469		1984			

Test for overall effect: Z = 4.89 (P < 0.00001)

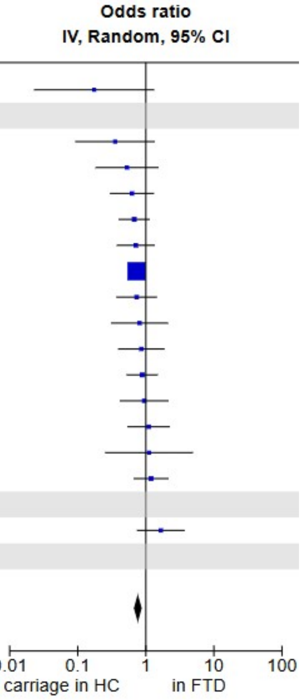

Heterogeneity: Tau^2 (REML[b]) = 0.00; Chi^2 = 12.48, df = 15 (P = 0.64); I^2 = 0%

Footnotes

[a]CI calculated by Wald-type method.

[b]Tau^2 calculated by Restricted Maximum-Likelihood method.

Figure S9 Funnel plot for the meta-analysis of *APOE2* carriage in frontotemporal dementia (FTD) vs. healthy controls (HC).

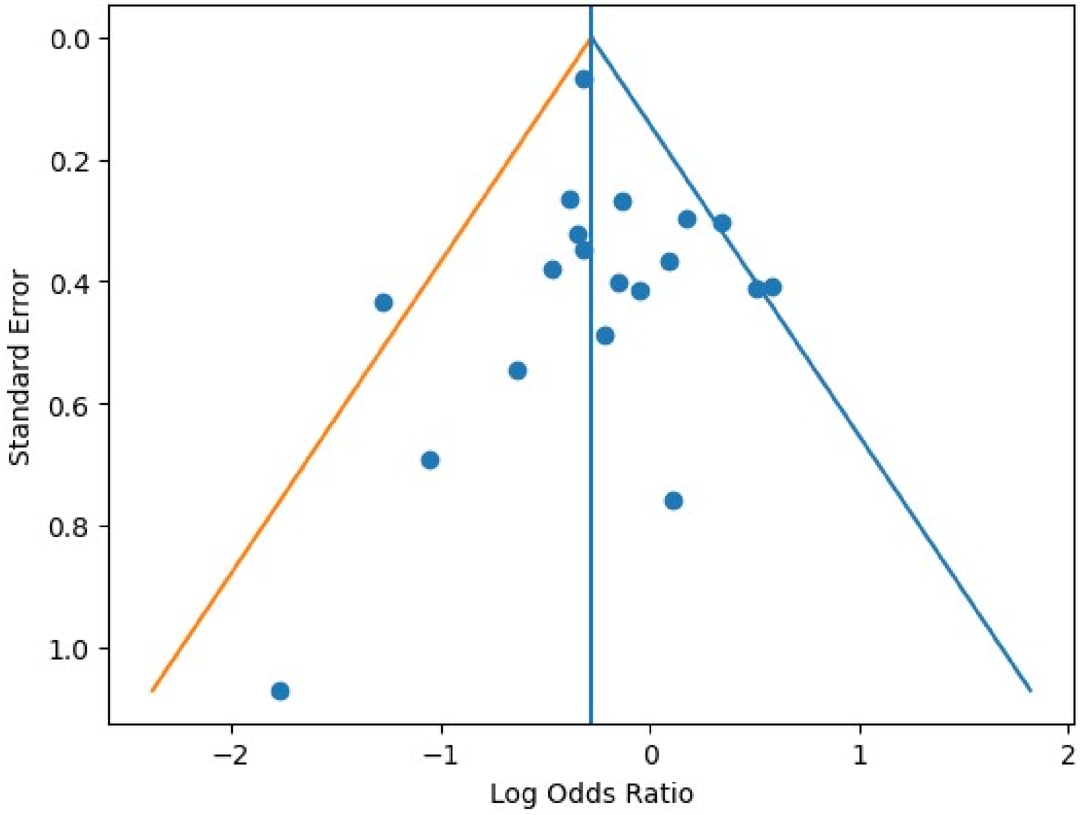

Figure S10 Meta-analyses of *APOE4* carriage status in corticobasal syndrome (CBS) vs. healthy controls (HC).

	CBS		HC			Odds ratio
**Study or Subgroup**	**Events**	**Total**	**Events**	**Total**	**Weight**	**IV, Random, 95% CI**
Galimberti (2009)	0	17	28	203	17.4%	0.18 [0.01 , 3.01]
Nasri (2022)	7	37	19	64	46.0%	0.55 [0.21 , 1.48]
Kurz (2025)	12	31	3	17	36.6%	2.95 [0.70 , 12.46]
**Total (Wald[a])**		**85**		**284**	**100.0%**	**0.84 [0.20 , 3.42]**
Total events:	19		50			

Test for overall effect: Z = 0.25 (P = 0.80)

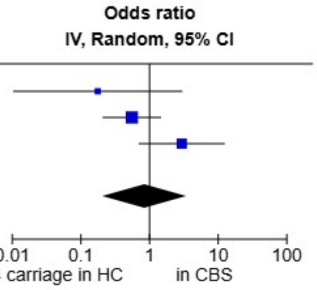

Heterogeneity: Tau² (REML[b]) = 0.87; Chi² = 4.78, df = 2 (P = 0.09); I² = 58%

Footnotes

[a]CI calculated by Wald-type method.

[b]Tau² calculated by Restricted Maximum-Likelihood method.

Figure S11 Exploratory meta-analyses of (a) *APOE4* carriage status in pathologically confirmed corticobasal degeneration (CBD) vs. healthy controls (HC) and (b) *APOE2* carriage status in pathologically confirmed CBD vs. HC.

	CBD		HC			Odds ratio
Study or Subgroup	Events	Total	Events	Total	Weight	IV, Random, 95% CI
Sabir (2019)	9	41	135	591	27.5%	0.95 [0.44 , 2.04]
Zhao (2018)	49	134	376	1406	72.5%	1.58 [1.09 , 2.29]
**Total (Wald[a])**		**175**		**1997**	**100.0%**	**1.37 [0.88 , 2.14]**
Total events:	58		511			

Test for overall effect: Z = 1.40 (P = 0.16)

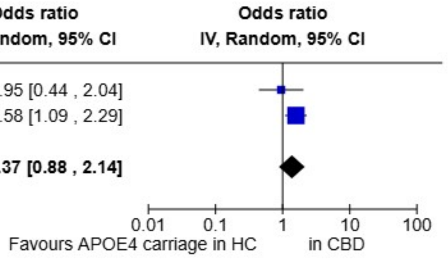

Heterogeneity: Tau² (REML[b]) = 0.04; Chi² = 1.38, df = 1 (P = 0.24); I² = 27%

Footnotes

[a]CI calculated by Wald-type method.

[b]Tau² calculated by Restricted Maximum-Likelihood method.

(a)

	CBD		HC			Odds ratio
Study or Subgroup	Events	Total	Events	Total	Weight	IV, Random, 95% CI
Sabir (2019)	5	41	102	591	20.9%	0.67 [0.26 , 1.74]
Zhao (2018)	20	134	261	1406	79.1%	0.77 [0.47 , 1.26]
**Total (Wald[a])**		**175**		**1997**	**100.0%**	**0.75 [0.48 , 1.16]**
Total events:	25		363			

Test for overall effect: Z = 1.30 (P = 0.19)

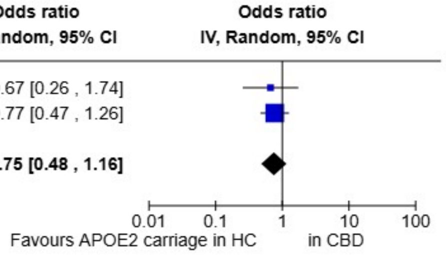

Heterogeneity: Tau² (REML[b]) = 0.00; Chi² = 0.07, df = 1 (P = 0.79); I² = 0%

Footnotes

[a]CI calculated by Wald-type method.

[b]Tau² calculated by Restricted Maximum-Likelihood method.

(b)

Figure S12 Meta-analyses of (a) *APOE4* carriage status in frontotemporal dementia with motor neuron disease (FTD-MND) vs. healthy controls (HC) and (b) *APOE2* carriage status in FTD-MND vs. HC.

(a)

Study or Subgroup	FTD-MND Events	FTD-MND Total	HC Events	HC Total	Weight	Odds ratio IV, Random, 95% CI
Chiò (2016)	7	52	30	223	8.3%	1.00 [0.41 , 2.42]
Srinivasan (2006)	12	30	201	756	11.5%	1.84 [0.87 , 3.89]
Mishra (2017)	63	293	1702	9400	80.2%	1.24 [0.93 , 1.65]
**Total (Wald[a])**		**375**		**10379**	**100.0%**	**1.27 [0.99 , 1.64]**
Total events:	82		1933			

Test for overall effect: Z = 1.87 (P = 0.06)

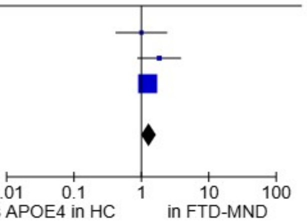

Heterogeneity: Tau² (REML[b]) = 0.00; Chi² = 1.25, df = 2 (P = 0.53); I² = 0%

Footnotes

[a]CI calculated by Wald-type method.

[b]Tau² calculated by Restricted Maximum-Likelihood method.

Study or Subgroup	FTD-MND Events	FTD-MND Total	HC Events	HC Total	Weight	Odds ratio IV, Random, 95% CI
Srinivasan (2006)	3	30	125	756	19.2%	0.56 [0.17 , 1.88]
Mishra (2017)	24	293	1250	9400	48.9%	0.58 [0.38 , 0.89]
Chiò (2016)	10	52	31	223	31.9%	1.47 [0.67 , 3.24]
**Total (Wald[a])**		**375**		**10379**	**100.0%**	**0.78 [0.41 , 1.47]**
Total events:	37		1406			

Test for overall effect: Z = 0.78 (P = 0.44)

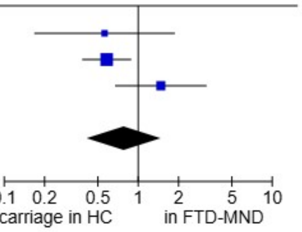

Heterogeneity: Tau² (REML[b]) = 0.17; Chi² = 4.31, df = 2 (P = 0.12); I² = 54%

Footnotes

[a]CI calculated by Wald-type method.

[b]Tau² calculated by Restricted Maximum-Likelihood method.

(b)

Figure S13 Meta-analysis of *APOE4* carriage status in behavioral variant frontotemporal dementia (bvFTD) vs. healthy controls (HC).

	bvFTD		HC			Odds ratio
**Study or Subgroup**	**Events**	**Total**	**Events**	**Total**	**Weight**	**IV, Random, 95% CI**
Mishra (2017)	457	2060	1702	9400	22.3%	1.29 [1.15 , 1.45]
Seripa (2012)	60	207	63	296	15.7%	1.51 [1.00 , 2.27]
Srinivasan (2006)	38	107	201	756	15.2%	1.52 [0.99 , 2.33]
Wang (2020)	10	30	8	33	5.2%	1.56 [0.52 , 4.69]
Agosta (2009)	8	31	10	56	5.6%	1.60 [0.56 , 4.60]
Borroni (2005)	18	51	10	50	7.0%	2.18 [0.89 , 5.37]
Galimberti (2009)	47	161	28	203	13.0%	2.58 [1.53 , 4.35]
Chen (2016)	10	28	185	1149	8.4%	2.89 [1.32 , 6.37]
Seripa (2011)	20	54	10	99	7.5%	5.24 [2.22 , 12.32]
**Total (Wald[a])**		**2729**		**12042**	**100.0%**	**1.87 [1.41 , 2.49]**
Total events:	668		2217			

Test for overall effect: Z = 4.29 (P < 0.0001)

Heterogeneity: Tau² (REML[b]) = 0.09; Chi² = 20.79, df = 8 (P = 0.008); I² = 62%

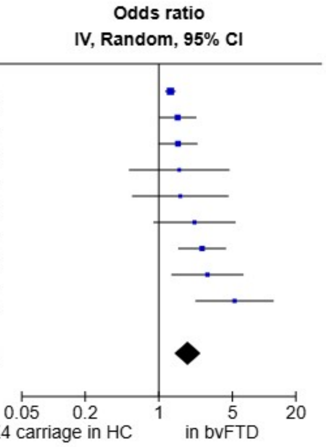

Footnotes

[a]CI calculated by Wald-type method.

[b]Tau² calculated by Restricted Maximum-Likelihood method.

Figure S14 Meta-analysis of *APOE4* carriage status in behavioral variant frontotemporal dementia (bvFTD) vs. Alzheimer's disease (AD).

	bvFTD		AD			Odds ratio	Odds ratio
**Study or Subgroup**	**Events**	**Total**	**Events**	**Total**	**Weight**	**IV, Random, 95% CI**	**IV, Random, 95% CI**
Agosta (2009)	8	31	30	51	29.4%	0.24 [0.09 , 0.65]	
Wang (2020)	10	30	28	63	32.3%	0.63 [0.25 , 1.55]	
Chen (2016)	10	28	433	1026	38.3%	0.76 [0.35 , 1.66]	
**Total (Wald[a])**		**89**		**1140**	**100.0%**	**0.51 [0.26 , 0.99]**	
Total events:	28		491				

Test for overall effect: Z = 2.00 (P = 0.05)

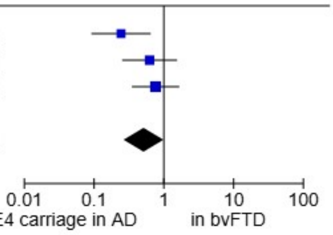

Heterogeneity: Tau² (REML[b]) = 0.14; Chi² = 3.37, df = 2 (P = 0.19); I² = 40%

Footnotes

[a]CI calculated by Wald-type method.

[b]Tau² calculated by Restricted Maximum-Likelihood method.

Figure S15 Meta-analysis of *APOE2* carriage status in behavioral variant frontotemporal dementia (bvFTD) vs. healthy controls (HC).

	bvFTD		HC			Odds ratio
Study or Subgroup	Events	Total	Events	Total	Weight	IV, Random, 95% CI
Chen (2016)	1	28	183	1149	0.5%	0.20 [0.03 , 1.45]
Agosta (2009)	3	31	13	56	1.1%	0.35 [0.09 , 1.36]
Seripa (2011)	6	54	17	99	2.0%	0.60 [0.22 , 1.63]
Srinivasan (2006)	13	107	125	756	5.3%	0.70 [0.38 , 1.29]
Seripa (2012)	18	207	34	296	5.5%	0.73 [0.40 , 1.34]
Mishra (2017)	214	2060	1250	9400	84.6%	0.76 [0.65 , 0.88]
Wang (2020)	4	30	4	33	0.9%	1.12 [0.25 , 4.92]
**Total (Wald[a])**		**2517**		**11789**	**100.0%**	**0.74 [0.64 , 0.85]**
Total events:	259		1626			

Test for overall effect: Z = 4.19 (P < 0.0001)

Odds ratio
IV, Random, 95% CI

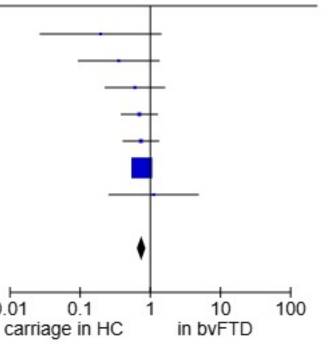

Heterogeneity: Tau² (REML[b]) = 0.00; Chi² = 3.42, df = 6 (P = 0.75); I² = 0%

Footnotes

[a]CI calculated by Wald-type method.

[b]Tau² calculated by Restricted Maximum-Likelihood method.

Figure S16 Meta-analysis of *APOE2* carriage status in behavioral variant frontotemporal dementia (bvFTD) vs. Alzheimer's disease (AD).

	bvFTD		AD			Odds ratio	Odds ratio
Study or Subgroup	Events	Total	Events	Total	Weight	IV, Random, 95% CI	IV, Random, 95% CI
Chen (2016)	1	28	90	1026	30.4%	0.39 [0.05 , 2.87]	
Wang (2020)	4	30	4	63	44.7%	2.27 [0.53 , 9.78]	
Agosta (2009)	3	31	1	51	24.9%	5.36 [0.53 , 53.97]	
**Total (Wald[a])**		**89**		**1140**	**100.0%**	**1.64 [0.43 , 6.26]**	
Total events:	8		95				

Test for overall effect: Z = 0.72 (P = 0.47)

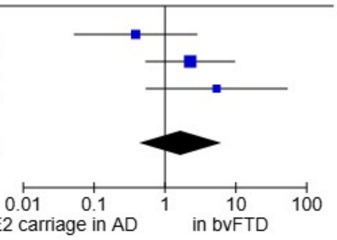

Heterogeneity: Tau² (REML[b]) = 0.49; Chi² = 3.20, df = 2 (P = 0.20); I² = 34%

Footnotes

[a]CI calculated by Wald-type method.

[b]Tau² calculated by Restricted Maximum-Likelihood method.

Figure S17 Meta-analysis of *APOE4* carriage status in semantic variant primary progressive aphasia (svPPA) vs. healthy controls (HC).

Study or Subgroup	svPPA Events	svPPA Total	HC Events	HC Total	Weight	Odds ratio IV, Random, 95% CI
Srinivasan (2006)	7	31	201	756	4.9%	0.81 [0.34 , 1.90]
Premi (2012)	7	28	41	200	4.2%	1.29 [0.51 , 3.25]
Chen (2016)	11	54	185	1149	7.8%	1.33 [0.67 , 2.63]
Mishra (2017)	119	496	1702	9400	79.8%	1.43 [1.15 , 1.77]
Borroni (2005)	10	27	10	50	3.3%	2.35 [0.83 , 6.68]
**Total (Wald[a])**		**636**		**11555**	**100.0%**	**1.40 [1.16 , 1.69]**
Total events:	154		2139			

Test for overall effect: Z = 3.46 (P = 0.0005)

Heterogeneity: Tau² (REML[b]) = 0.00; Chi² = 2.63, df = 4 (P = 0.62); I² = 0%

Odds ratio IV, Random, 95% CI

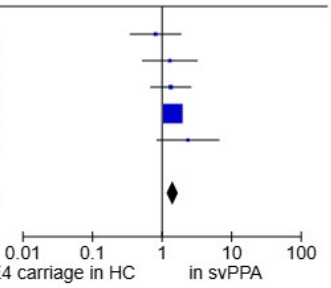

Footnotes

[a]CI calculated by Wald-type method.

[b]Tau² calculated by Restricted Maximum-Likelihood method.

Figure S18 Meta-analysis of *APOE4* carriage status in semantic variant primary progressive aphasia (svPPA) vs. Alzheimer's disease (AD).

	svPPA		AD			Odds ratio	Odds ratio
Study or Subgroup	Events	Total	Events	Total	Weight	IV, Random, 95% CI	IV, Random, 95% CI
Chen (2016)	11	54	433	1026	100.0%	0.35 [0.18 , 0.69]	
**Total**		**54**		**1026**	**100.0%**	**0.35 [0.18 , 0.69]**	
Total events:	11		433				

Test for overall effect: Z = 3.05 (P = 0.002)

Heterogeneity: Not applicable

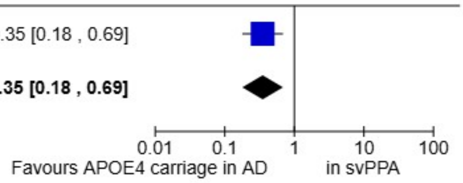

Figure S19 Meta-analysis of *APOE2* carriage status in semantic variant primary progressive aphasia (svPPA) vs. healthy controls (HC).

	svPPA		HC			Odds ratio
**Study or Subgroup**	**Events**	**Total**	**Events**	**Total**	**Weight**	**IV, Random, 95% CI**
Mishra (2017)	46	496	1250	9400	76.8%	0.67 [0.49 , 0.91]
Srinivasan (2006)	4	31	125	756	6.4%	0.75 [0.26 , 2.17]
Premi (2012)	2	28	15	200	3.1%	0.95 [0.21 , 4.39]
Chen (2016)	9	54	183	1149	13.7%	1.06 [0.51 , 2.20]
**Total (Wald[a])**		**609**		**11505**	**100.0%**	**0.72 [0.55 , 0.95]**
Total events:	61		1573			

Test for overall effect: Z = 2.35 (P = 0.02)

Odds ratio
IV, Random, 95% CI

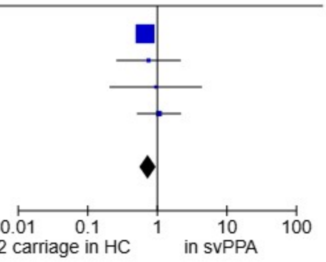

Heterogeneity: Tau² (REML[b]) = 0.00; Chi² = 1.42, df = 3 (P = 0.70); I² = 0%

Footnotes

[a]CI calculated by Wald-type method.

[b]Tau² calculated by Restricted Maximum-Likelihood method.

Figure S20 Meta-analysis of *APOE2* carriage status in semantic variant primary progressive aphasia (svPPA) vs. Alzheimer's disease (AD).

	svPPA		AD			Odds ratio
Study or Subgroup	Events	Total	Events	Total	Weight	IV, Random, 95% CI
Chen (2016)	9	54	90	1026	100.0%	2.08 [0.98 , 4.39]
**Total**		**54**		**1026**	**100.0%**	**2.08 [0.98 , 4.39]**
Total events:	9		90			

Test for overall effect: Z = 1.92 (P = 0.05)

Heterogeneity: Not applicable

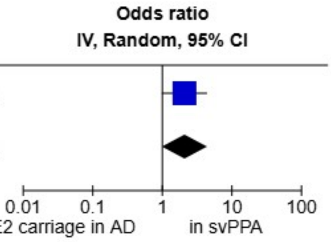

Figure S21 Meta-analysis of *APOE4* carriage status in non-fluent variant primary progressive aphasia (nfvPPA) vs. healthy controls (HC).

	nfvPPA		HC			Odds ratio	Odds ratio
**Study or Subgroup**	**Events**	**Total**	**Events**	**Total**	**Weight**	**IV, Random, 95% CI**	**IV, Random, 95% CI**
Mishra (2017)	106	490	1702	9400	72.2%	1.25 [1.00 , 1.56]	
Premi (2012)	22	66	41	200	27.8%	1.94 [1.05 , 3.59]	
**Total (Wald[a])**		**556**		**9600**	**100.0%**	**1.41 [0.96 , 2.08]**	
Total events:	128		1743				

Test for overall effect: Z = 1.75 (P = 0.08)

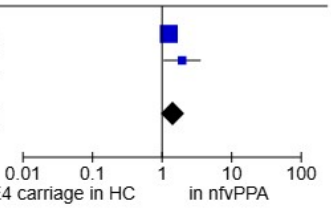

Heterogeneity: Tau² (REML[b]) = 0.04; Chi² = 1.74, df = 1 (P = 0.19); I² = 42%

Footnotes

[a]CI calculated by Wald-type method.

[b]Tau² calculated by Restricted Maximum-Likelihood method.

Figure S22 Meta-analysis of *APOE2* carriage status in non-fluent variant primary progressive aphasia (nfvPPA) vs. healthy controls (HC).

	nfvPPA		HC			Odds ratio
Study or Subgroup	Events	Total	Events	Total	Weight	IV, Random, 95% CI
Mishra (2017)	47	490	1250	9400	56.2%	0.69 [0.51 , 0.94]
Premi (2012)	10	66	15	200	43.8%	2.20 [0.94 , 5.17]
**Total (Wald[a])**		**556**		**9600**	**100.0%**	**1.15 [0.37 , 3.54]**
Total events:	57		1265			

Test for overall effect: Z = 0.24 (P = 0.81)

Heterogeneity: Tau² (REML[b]) = 0.56; Chi² = 6.26, df = 1 (P = 0.01); I² = 84%

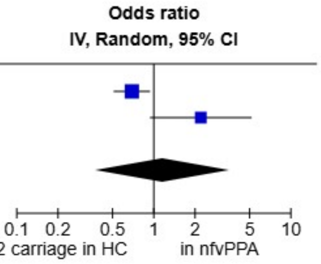

Footnotes

[a]CI calculated by Wald-type method.

[b]Tau² calculated by Restricted Maximum-Likelihood method.

Figure S23 Sensitivity analysis of *APOE4* carriage status in frontotemporal dementia (FTD) vs. healthy controls (HC), based on study quality.

Study or Subgroup	Events	Total	Events	Total	Weight	Odds ratio IV, Random, 95% CI
✓ Verpillat (2002)	18	94	114	392	6.0%	0.58 [0.33 , 1.01]
✕ Kowalska (2001)	3	24	25	200	0.0%	1.00 [0.28 , 3.60]
✓ Riemenschneider (2002)	10	52	35	182	4.6%	1.00 [0.46 , 2.19]
✓ Rogalski (2011)	42	149	89	330	6.9%	1.06 [0.69 , 1.64]
✓ Villa (2014)	60	290	63	341	7.1%	1.15 [0.78 , 1.71]
✓ Mishra (2017)	682	3046	1702	9400	8.6%	1.30 [1.18 , 1.44]
✕ Albani (2008)	14	73	23	151	0.0%	1.32 [0.63 , 2.75]
✓ Srinivasan (2006)	53	161	201	765	7.3%	1.38 [0.95 , 1.99]
✕ Sorrentino (2023)	59	217	51	240	0.0%	1.38 [0.90 , 2.13]
✕ Seripa (2012)	83	282	63	296	0.0%	1.54 [1.06 , 2.25]
✕ Daniele (2009)	44	127	87	343	0.0%	1.56 [1.01 , 2.42]
✕ Wang (2020)	10	30	8	33	0.0%	1.56 [0.52 , 4.69]
✓ Lovati (2010)	17	75	79	506	5.8%	1.58 [0.88 , 2.86]
✓ Agosta (2009)	8	31	10	56	3.4%	1.60 [0.56 , 4.60]
✓ Morenas-Rodríguez (2016)	11	32	18	74	4.0%	1.63 [0.66 , 4.02]
✕ Nielsen (2003)	17	49	39	163	0.0%	1.69 [0.85 , 3.37]
✓ Ruiz (2014)	64	261	257	1596	7.6%	1.69 [1.24 , 2.31]
✕ Premi (2012)	29	94	41	200	0.0%	1.73 [0.99 , 3.02]
✓ Chen (2016)	21	82	185	1149	6.3%	1.79 [1.07 , 3.02]
✕ Johansson (2003)	26	63	43	153	0.0%	1.80 [0.97 , 3.32]
✓ Borroni (2005)	32	86	10	50	4.4%	2.37 [1.04 , 5.38]
✓ Galimberti (2009)	52	176	28	203	6.3%	2.62 [1.57 , 4.38]
✓ Bernardi (2006)	34	100	29	180	5.9%	2.68 [1.51 , 4.76]
✓ Ji (2013)	20	62	55	381	5.7%	2.82 [1.54 , 5.17]
✓ Venturelli (2008)	21	71	22	190	5.3%	3.21 [1.63 , 6.31]
✕ Sadhukhan (2023)	46	122	38	256	0.0%	3.47 [2.10 , 5.74]
✕ Fabre (2001)	33	64	10	47	0.0%	3.94 [1.68 , 9.25]
✓ Seripa (2011)	36	86	10	99	4.7%	6.41 [2.93 , 14.00]
**Total (Wald[a])**		**4854**		**15894**	**100.0%**	**1.70 [1.33 , 2.18]**
Total events:	1181		2907			

Test for overall effect: Z = 4.21 (P < 0.0001)

Odds ratio IV, Random, 95% CI

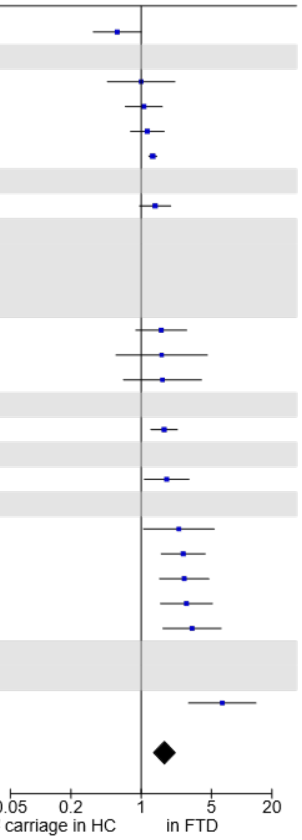

Heterogeneity: Tau² (REML[b]) = 0.18; Chi² = 55.11, df = 16 (P < 0.00001); I² = 80%

Footnotes

[a]CI calculated by Wald-type method.

[b]Tau² calculated by Restricted Maximum-Likelihood method.

Figure S24 Sensitivity analysis of *APOE4* carriage status in frontotemporal dementia (FTD) vs. Alzheimer's disease (AD), based on study quality.

Study or Subgroup	Events	Total	Events	Total	Weight	Odds ratio IV, Random, 95% CI
✕ Sadhukhan (2023)	46	122	78	94	0.0%	0.12 [0.06 , 0.24]
✓ Rogalski (2011)	42	149	116	179	10.9%	0.21 [0.13 , 0.34]
✕ Kowalska (2001)	3	24	26	65	0.0%	0.21 [0.06 , 0.79]
✓ Agosta (2009)	8	31	30	51	3.3%	0.24 [0.09 , 0.65]
✕ Nielsen (2003)	17	49	207	320	0.0%	0.29 [0.15 , 0.55]
✓ Sorrentino (2023)	59	217	124	221	13.5%	0.29 [0.20 , 0.44]
✓ Villa (2014)	60	290	160	352	15.7%	0.31 [0.22 , 0.45]
✕ Engelborghs (2003)	14	47	281	504	0.0%	0.34 [0.18 , 0.64]
✓ Morenas-Rodríguez (2016)	11	32	44	73	4.1%	0.35 [0.15 , 0.82]
✓ Lovati (2010)	17	75	323	735	8.4%	0.37 [0.21 , 0.65]
✓ Ruiz (2014)	64	261	1264	2821	19.2%	0.40 [0.30 , 0.54]
✕ Johansson (2003)	26	63	173	272	0.0%	0.40 [0.23 , 0.70]
✓ Kaivorinne (2008)	25	59	77	122	6.9%	0.43 [0.23 , 0.81]
✓ Chen (2016)	21	82	433	1026	9.7%	0.47 [0.28 , 0.79]
✓ Ji (2013)	20	62	189	432	8.3%	0.61 [0.35 , 1.08]
✕ Wang (2020)	10	30	28	63	0.0%	0.63 [0.25 , 1.55]
✕ Sakurai (2024)	17	44	59	118	0.0%	0.63 [0.31 , 1.28]
**Total (Wald[a])**		**1258**		**6012**	**100.0%**	**0.35 [0.29 , 0.43]**
Total events:	327		2760			
Test for overall effect: Z = 10.97 (P < 0.00001)						

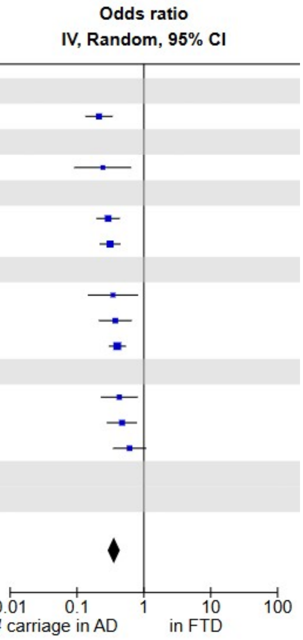

Heterogeneity: Tau² (REML[b]) = 0.02; Chi² = 12.27, df = 9 (P = 0.20); I² = 29%

Footnotes

[a]CI calculated by Wald-type method.

[b]Tau² calculated by Restricted Maximum-Likelihood method.

Figure S25 Sensitivity analysis of *APOE2* carriage status in frontotemporal dementia (FTD) vs. healthy controls (HC), based on study quality.

Study or Subgroup	Events	Total	Events	Total	Weight	Odds ratio IV, Random, 95% CI
✕ Kowalska (2001)	1	24	40	200	0.0%	0.17 [0.02 , 1.33]
✓ Bernardi (2006)	7	100	38	180	1.8%	0.28 [0.12 , 0.66]
✓ Agosta (2009)	3	31	13	56	0.7%	0.35 [0.09 , 1.36]
✓ Ji (2013)	4	62	44	381	1.2%	0.53 [0.18 , 1.53]
✕ Sadhukhan (2023)	10	122	32	256	0.0%	0.63 [0.30 , 1.32]
✓ Srinivasan (2006)	19	161	125	756	4.9%	0.68 [0.40 , 1.13]
✓ Rogalski (2011)	14	149	42	330	3.2%	0.71 [0.38 , 1.35]
✓ Mishra (2017)	307	3046	1250	9400	74.8%	0.73 [0.64 , 0.83]
✓ Chen (2016)	10	82	183	1149	2.8%	0.73 [0.37 , 1.45]
✕ Nielsen (2003)	6	49	24	163	0.0%	0.81 [0.31 , 2.11]
✓ Seripa (2011)	13	86	17	99	2.1%	0.86 [0.39 , 1.89]
✕ Seripa (2012)	29	282	34	296	0.0%	0.88 [0.52 , 1.49]
✓ Riemenschneider (2002)	9	52	33	182	2.0%	0.95 [0.42 , 2.13]
✓ Lovati (2010)	10	75	62	506	2.5%	1.10 [0.54 , 2.26]
✕ Wang (2020)	4	30	4	33	0.0%	1.12 [0.25 , 4.92]
✓ Verpillat (2002)	18	94	65	392	3.9%	1.19 [0.67 , 2.13]
✕ Daniele (2009)	19	127	38	343	0.0%	1.41 [0.78 , 2.55]
✕ Albani (2008)	12	73	16	151	0.0%	1.66 [0.74 , 3.72]
✕ Premi (2012)	12	94	15	200	0.0%	1.80 [0.81 , 4.03]
**Total (Wald[a])**		**3938**		**13431**	**100.0%**	**0.74 [0.66 , 0.83]**
Total events:	414		1872			

Test for overall effect: Z = 5.26 (P < 0.00001)

Heterogeneity: Tau² (REML[b]) = 0.00; Chi² = 10.98, df = 10 (P = 0.36); I² = 0%

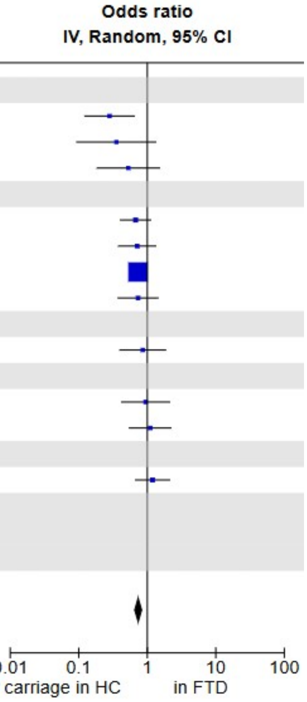

Footnotes

[a]CI calculated by Wald-type method.

[b]Tau² calculated by Restricted Maximum-Likelihood method.

Figure S26 Sensitivity analysis of *APOE2* carriage status in frontotemporal dementia (FTD) vs. Alzheimer's disease (AD), based on study quality.

Study or Subgroup	Events	Total	Events	Total	Weight	Odds ratio IV, Random, 95% CI
✕ Kowalska (2001)	1	24	3	65	0.0%	0.90 [0.09 , 9.08]
✓ Ji (2013)	4	62	26	432	13.2%	1.08 [0.36 , 3.20]
✓ Chen (2016)	10	82	90	1026	32.2%	1.44 [0.72 , 2.90]
✓ Rogalski (2011)	14	149	11	179	23.1%	1.58 [0.70 , 3.60]
✕ Nielsen (2003)	6	49	21	320	0.0%	1.99 [0.76 , 5.20]
✕ Sadhukhan (2023)	10	122	4	94	0.0%	2.01 [0.61 , 6.62]
✕ Wang (2020)	4	30	4	63	0.0%	2.27 [0.53 , 9.78]
✓ Lovati (2010)	10	75	40	735	28.6%	2.67 [1.28 , 5.59]
✓ Agosta (2009)	3	31	1	51	2.9%	5.36 [0.53 , 53.97]
**Total (Wald[a])**		**399**		**2423**	**100.0%**	**1.76 [1.19 , 2.61]**
Total events:	41		168			

Test for overall effect: Z = 2.80 (P = 0.005)

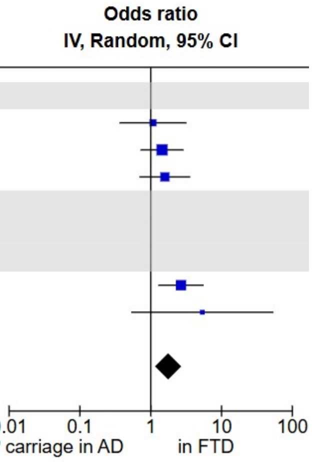

Heterogeneity: Tau^2 (REML[b]) = 0.00; Chi^2 = 3.28, df = 4 (P = 0.51); I^2 = 0%

Footnotes

[a]CI calculated by Wald-type method.

[b]Tau^2 calculated by Restricted Maximum-Likelihood method.

Figure S27 Sensitivity analysis of *APOE4* carriage status in progressive supranuclear palsy (PSP) vs. healthy controls (HC), based on study quality.

Study or Subgroup	Events	Total	Events	Total	Weight	Odds ratio IV, Random, 95% CI
✓ Wang (2024)	57	273	905	2818	37.8%	0.56 [0.41 , 0.75]
✓ Xiao (2022)	11	104	84	488	20.6%	0.57 [0.29 , 1.11]
✕ Morris (2000)	9	48	18	65	0.0%	0.60 [0.24 , 1.49]
✓ Anouti (1996)	13	52	14	52	14.6%	0.90 [0.38 , 2.18]
✓ Nasri (2022)	20	58	19	64	17.6%	1.25 [0.58 , 2.67]
✓ Galimberti (2009)	4	19	28	203	9.4%	1.67 [0.52 , 5.39]
**Total (Wald[a])**		**506**		**3625**	**100.0%**	**0.77 [0.51 , 1.15]**
Total events:	105		1050			

Test for overall effect: Z = 1.30 (P = 0.20)

Heterogeneity: Tau² (REML[b]) = 0.09; Chi² = 6.95, df = 4 (P = 0.14); I² = 44%

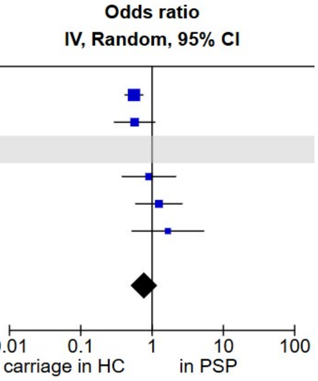

Footnotes

[a]CI calculated by Wald-type method.

[b]Tau² calculated by Restricted Maximum-Likelihood method.

Figure S28 Sensitivity analysis of APOE2 carriage status in progressive supranuclear palsy (PSP) vs. healthy controls (HC), based on study quality.

Study or Subgroup	Events	Total	Events	Total	Weight	Odds ratio IV, Random, 95% CI
✓ Xiao (2022)	12	104	79	488	30.3%	0.68 [0.35 , 1.29]
✓ Anouti (1996)	9	52	7	52	17.5%	1.35 [0.46 , 3.93]
✕ Morris (2000)	6	48	6	65	0.0%	1.40 [0.42 , 4.66]
✓ Nasri (2022)	4	58	3	64	10.2%	1.51 [0.32 , 7.03]
✓ Wang (2024)	36	274	220	2742	42.0%	1.73 [1.19 , 2.53]
**Total (Wald[a])**		**488**		**3346**	**100.0%**	**1.23 [0.71 , 2.13]**
Total events:	61		309			

Test for overall effect: Z = 0.73 (P = 0.46)

Odds ratio IV, Random, 95% CI

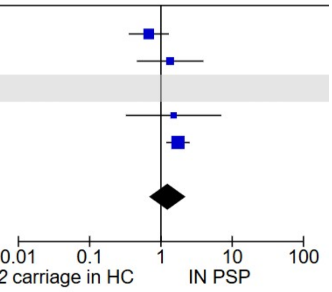

Heterogeneity: Tau² (REML[b]) = 0.15; Chi² = 6.10, df = 3 (P = 0.11); I² = 51%

Footnotes

[a]CI calculated by Wald-type method.

[b]Tau² calculated by Restricted Maximum-Likelihood method.

Figure S29 Exploratory meta-analysis of *APOE* ε4 carriage in frontotemporal dementia (FTD) vs. healthy controls (HC) among studies with a European/White population background.

Study or Subgroup	Events	Total	Events	Total	Weight	Odds ratio IV, Random, 95% CI
✓ Verpillat (2002)	18	94	114	392	7.7%	0.58 [0.33 , 1.01]
✕ Kowalska (2001)	3	24	25	200	0.0%	1.00 [0.28 , 3.60]
✓ Riemenschneider (2002)	10	52	35	182	6.1%	1.00 [0.46 , 2.19]
✕ Rogalski (2011)	42	149	89	330	0.0%	1.06 [0.69 , 1.64]
✓ Villa (2014)	60	290	63	341	9.0%	1.15 [0.78 , 1.71]
✓ Mishra (2017)	682	3046	1702	9400	10.6%	1.30 [1.18 , 1.44]
✓ Albani (2008)	14	73	23	151	6.4%	1.32 [0.63 , 2.75]
✓ Srinivasan (2006)	53	161	201	765	9.2%	1.38 [0.95 , 1.99]
✕ Sorrentino (2023)	59	217	51	240	0.0%	1.38 [0.90 , 2.13]
✓ Seripa (2012)	83	282	63	296	9.1%	1.54 [1.06 , 2.25]
✕ Daniele (2009)	44	127	87	343	0.0%	1.56 [1.01 , 2.42]
✕ Wang (2020)	10	30	8	33	0.0%	1.56 [0.52 , 4.69]
✕ Lovati (2010)	17	75	79	506	0.0%	1.58 [0.88 , 2.86]
✕ Agosta (2009)	8	31	10	56	0.0%	1.60 [0.56 , 4.60]
✕ Morenas-Rodríguez (2016)	11	32	18	74	0.0%	1.63 [0.66 , 4.02]
✕ Nielsen (2003)	17	49	39	163	0.0%	1.69 [0.85 , 3.37]
✓ Ruiz (2014)	64	261	257	1596	9.5%	1.69 [1.24 , 2.31]
✕ Premi (2012)	29	94	41	200	0.0%	1.73 [0.99 , 3.02]
✕ Chen (2016)	21	82	185	1149	0.0%	1.79 [1.07 , 3.02]
✓ Johansson (2003)	26	63	43	153	7.3%	1.80 [0.97 , 3.32]
✓ Borroni (2005)	32	86	10	50	5.8%	2.37 [1.04 , 5.38]
✕ Galimberti (2009)	52	176	28	203	0.0%	2.62 [1.57 , 4.38]
✓ Bernardi (2006)	34	100	29	180	7.6%	2.68 [1.51 , 4.76]
✕ Ji (2013)	20	62	55	381	0.0%	2.82 [1.54 , 5.17]
✕ Venturelli (2008)	21	71	22	190	0.0%	3.21 [1.63 , 6.31]
✕ Sadhukhan (2023)	46	122	38	256	0.0%	3.47 [2.10 , 5.74]
✓ Fabre (2001)	33	64	10	47	5.6%	3.94 [1.68 , 9.25]
✓ Seripa (2011)	36	86	10	99	6.1%	6.41 [2.93 , 14.00]
**Total (Wald[a])**		**4658**		**13652**	**100.0%**	**1.64 [1.22 , 2.20]**
Total events:	1145		2560			
Test for overall effect: Z = 3.29 (P = 0.0010)						

Odds ratio
IV, Random, 95% CI

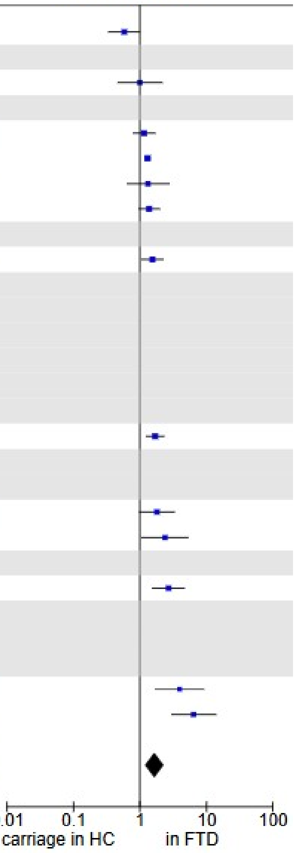

Heterogeneity: Tau² (REML[b]) = 0.21; Chi² = 42.19, df = 12 (P < 0.0001); I² = 84%

Footnotes

[a]CI calculated by Wald-type method.

[b]Tau² calculated by Restricted Maximum-Likelihood method.

Figure S30 Exploratory meta-analysis of *APOE* ε4 carriage in frontotemporal dementia (FTD) vs. healthy controls (HC) among East Asian studies.

Study or Subgroup	Events	Total	Events	Total	Weight	Odds ratio IV, Random, 95% CI
✕ Verpillat (2002)	18	94	114	392	0.0%	0.58 [0.33 , 1.01]
✓ Kowalska (2001)	3	24	25	200	7.8%	1.00 [0.28 , 3.60]
✕ Riemenschneider (2002)	10	52	35	182	0.0%	1.00 [0.46 , 2.19]
✕ Rogalski (2011)	42	149	89	330	0.0%	1.06 [0.69 , 1.64]
✕ Villa (2014)	60	290	63	341	0.0%	1.15 [0.78 , 1.71]
✕ Mishra (2017)	682	3046	1702	9400	0.0%	1.30 [1.18 , 1.44]
✕ Albani (2008)	14	73	23	151	0.0%	1.32 [0.63 , 2.75]
✕ Srinivasan (2006)	53	161	201	765	0.0%	1.38 [0.95 , 1.99]
✕ Sorrentino (2023)	59	217	51	240	0.0%	1.38 [0.90 , 2.13]
✕ Seripa (2012)	83	282	63	296	0.0%	1.54 [1.06 , 2.25]
✕ Daniele (2009)	44	127	87	343	0.0%	1.56 [1.01 , 2.42]
✓ Wang (2020)	10	30	8	33	10.5%	1.56 [0.52 , 4.69]
✕ Lovati (2010)	17	75	79	506	0.0%	1.58 [0.88 , 2.86]
✕ Agosta (2009)	8	31	10	56	0.0%	1.60 [0.56 , 4.60]
✕ Morenas-Rodríguez (2016)	11	32	18	74	0.0%	1.63 [0.66 , 4.02]
✕ Nielsen (2003)	17	49	39	163	0.0%	1.69 [0.85 , 3.37]
✕ Ruiz (2014)	64	261	257	1596	0.0%	1.69 [1.24 , 2.31]
✕ Premi (2012)	29	94	41	200	0.0%	1.73 [0.99 , 3.02]
✓ Chen (2016)	21	82	185	1149	46.9%	1.79 [1.07 , 3.02]
✕ Johansson (2003)	26	63	43	153	0.0%	1.80 [0.97 , 3.32]
✕ Borroni (2005)	32	86	10	50	0.0%	2.37 [1.04 , 5.38]
✕ Galimberti (2009)	52	176	28	203	0.0%	2.62 [1.57 , 4.38]
✕ Bernardi (2006)	34	100	29	180	0.0%	2.68 [1.51 , 4.76]
✓ Ji (2013)	20	62	55	381	34.8%	2.82 [1.54 , 5.17]
✕ Venturelli (2008)	21	71	22	190	0.0%	3.21 [1.63 , 6.31]
✕ Sadhukhan (2023)	46	122	38	256	0.0%	3.47 [2.10 , 5.74]
✕ Fabre (2001)	33	64	10	47	0.0%	3.94 [1.68 , 9.25]
✕ Seripa (2011)	36	86	10	99	0.0%	6.41 [2.93 , 14.00]
**Total (Wald[a])**		**198**		**1763**	**100.0%**	**1.98 [1.39 , 2.83]**
Total events:	54		273			

Test for overall effect: Z = 3.75 (P = 0.0002)

Heterogeneity: Tau² (REML[b]) = 0.00; Chi² = 2.73, df = 3 (P = 0.43); I² = 0%

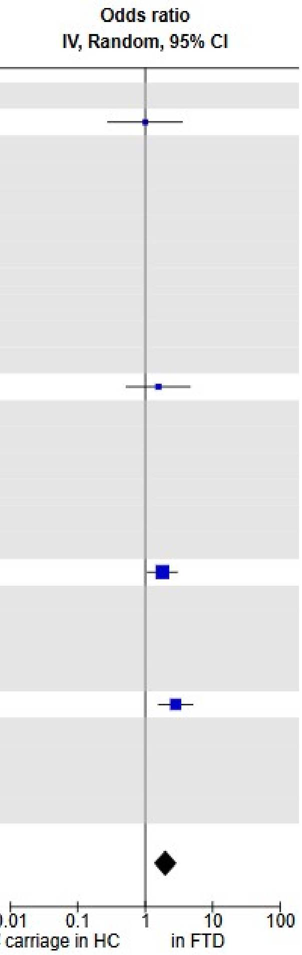

Footnotes

[a]CI calculated by Wald-type method.

[b]Tau² calculated by Restricted Maximum-Likelihood method.

Figure S31 Exploratory meta-analysis of *APOE4* carriage in frontotemporal dementia (FTD) vs. Alzheimer's disease (AD) among studies with a European/White population background.

Study or Subgroup	Events	Total	Events	Total	Weight	Odds ratio IV, Random, 95% CI
✕ Sadhukhan (2023)	46	122	78	94	0.0%	0.12 [0.06 , 0.24]
✕ Rogalski (2011)	42	149	116	179	0.0%	0.21 [0.13 , 0.34]
✕ Kowalska (2001)	3	24	26	65	0.0%	0.21 [0.06 , 0.79]
✕ Agosta (2009)	8	31	30	51	0.0%	0.24 [0.09 , 0.65]
✕ Nielsen (2003)	17	49	207	320	0.0%	0.29 [0.15 , 0.55]
✕ Sorrentino (2023)	59	217	124	221	0.0%	0.29 [0.20 , 0.44]
✓ Villa (2014)	60	290	160	352	28.8%	0.31 [0.22 , 0.45]
✓ Engelborghs (2003)	14	47	281	504	8.5%	0.34 [0.18 , 0.64]
✕ Morenas-Rodríguez (2016)	11	32	44	73	0.0%	0.35 [0.15 , 0.82]
✕ Lovati (2010)	17	75	323	735	0.0%	0.37 [0.21 , 0.65]
✓ Ruiz (2014)	64	261	1264	2821	42.3%	0.40 [0.30 , 0.54]
✓ Johansson (2003)	26	63	173	272	11.5%	0.40 [0.23 , 0.70]
✓ Kaivorinne (2008)	25	59	77	122	8.9%	0.43 [0.23 , 0.81]
✕ Chen (2016)	21	82	433	1026	0.0%	0.47 [0.28 , 0.79]
✕ Ji (2013)	20	62	189	432	0.0%	0.61 [0.35 , 1.08]
✕ Wang (2020)	10	30	28	63	0.0%	0.63 [0.25 , 1.55]
✕ Sakurai (2024)	17	44	59	118	0.0%	0.63 [0.31 , 1.28]
**Total (Wald[a])**		**720**		**4071**	**100.0%**	**0.37 [0.31 , 0.45]**
Total events:	189		1955			

Test for overall effect: Z = 10.28 (P < 0.00001)

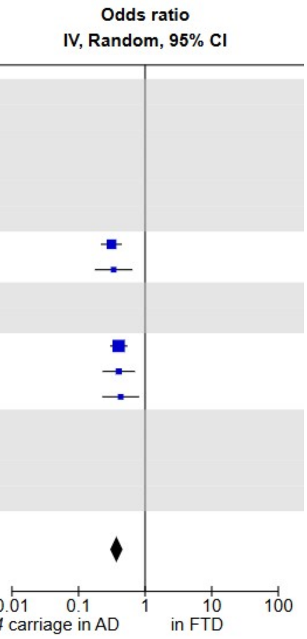

Heterogeneity: Tau² (REML[b]) = 0.00; Chi² = 1.52, df = 4 (P = 0.82); I² = 0%

Footnotes

[a]CI calculated by Wald-type method.

[b]Tau² calculated by Restricted Maximum-Likelihood method.

Figure S32 Exploratory meta-analysis of *APOE4* carriage in frontotemporal dementia (FTD) vs. Alzheimer's disease (AD) among East Asian studies.

Study or Subgroup	Events	Total	Events	Total	Weight	Odds ratio IV, Random, 95% CI
✕ Sadhukhan (2023)	46	122	78	94	0.0%	0.12 [0.06 , 0.24]
✕ Rogalski (2011)	42	149	116	179	0.0%	0.21 [0.13 , 0.34]
✓ Kowalska (2001)	3	24	26	65	6.7%	0.21 [0.06 , 0.79]
✕ Agosta (2009)	8	31	30	51	0.0%	0.24 [0.09 , 0.65]
✕ Nielsen (2003)	17	49	207	320	0.0%	0.29 [0.15 , 0.55]
✕ Sorrentino (2023)	59	217	124	221	0.0%	0.29 [0.20 , 0.44]
✕ Villa (2014)	60	290	160	352	0.0%	0.31 [0.22 , 0.45]
✕ Engelborghs (2003)	14	47	281	504	0.0%	0.34 [0.18 , 0.64]
✕ Morenas-Rodríguez (2016)	11	32	44	73	0.0%	0.35 [0.15 , 0.82]
✕ Lovati (2010)	17	75	323	735	0.0%	0.37 [0.21 , 0.65]
✕ Ruiz (2014)	64	261	1264	2821	0.0%	0.40 [0.30 , 0.54]
✕ Johansson (2003)	26	63	173	272	0.0%	0.40 [0.23 , 0.70]
✕ Kaivorinne (2008)	25	59	77	122	0.0%	0.43 [0.23 , 0.81]
✓ Chen (2016)	21	82	433	1026	43.7%	0.47 [0.28 , 0.79]
✓ Ji (2013)	20	62	189	432	35.7%	0.61 [0.35 , 1.08]
✓ Wang (2020)	10	30	28	63	13.9%	0.63 [0.25 , 1.55]
✕ Sakurai (2024)	17	44	59	118	0.0%	0.63 [0.31 , 1.28]
**Total (Wald[a])**		**198**		**1586**	**100.0%**	**0.51 [0.36 , 0.72]**
Total events:	54		676			

Test for overall effect: Z = 3.90 (P < 0.0001)

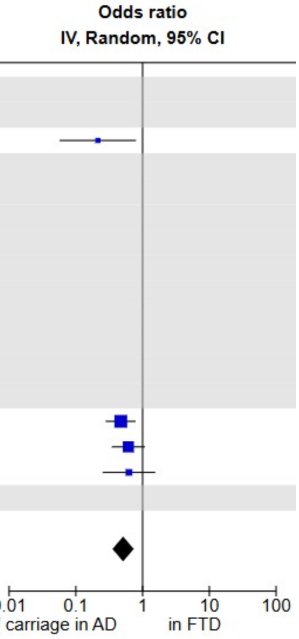

Heterogeneity: Tau² (REML[b]) = 0.00; Chi² = 2.37, df = 3 (P = 0.50); I² = 0%

Footnotes

[a]CI calculated by Wald-type method.

[b]Tau² calculated by Restricted Maximum-Likelihood method.

Figure S33 Exploratory meta-analysis of *APOE* ε2 carriage in frontotemporal dementia (FTD) vs. healthy controls (HC) among studies with a European/White population background.

Study or Subgroup	Events	Total	Events	Total	Weight	Odds ratio IV, Random, 95% CI
✕ Kowalska (2001)	1	24	40	200	0.0%	0.17 [0.02 , 1.33]
✓ Bernardi (2006)	7	100	38	180	5.5%	0.28 [0.12 , 0.66]
✕ Agosta (2009)	3	31	13	56	0.0%	0.35 [0.09 , 1.36]
✕ Ji (2013)	4	62	44	381	0.0%	0.53 [0.18 , 1.53]
✕ Sadhukhan (2023)	10	122	32	256	0.0%	0.63 [0.30 , 1.32]
✓ Srinivasan (2006)	19	161	125	756	12.5%	0.68 [0.40 , 1.13]
✕ Rogalski (2011)	14	149	42	330	0.0%	0.71 [0.38 , 1.35]
✓ Mishra (2017)	307	3046	1250	9400	41.4%	0.73 [0.64 , 0.83]
✕ Chen (2016)	10	82	183	1149	0.0%	0.73 [0.37 , 1.45]
✕ Nielsen (2003)	6	49	24	163	0.0%	0.81 [0.31 , 2.11]
✓ Seripa (2011)	13	86	17	99	6.2%	0.86 [0.39 , 1.89]
✓ Seripa (2012)	29	282	34	296	12.1%	0.88 [0.52 , 1.49]
✓ Riemenschneider (2002)	9	52	33	182	5.9%	0.95 [0.42 , 2.13]
✕ Lovati (2010)	10	75	62	506	0.0%	1.10 [0.54 , 2.26]
✕ Wang (2020)	4	30	4	33	0.0%	1.12 [0.25 , 4.92]
✓ Verpillat (2002)	18	94	65	392	10.4%	1.19 [0.67 , 2.13]
✕ Daniele (2009)	19	127	38	343	0.0%	1.41 [0.78 , 2.55]
✓ Albani (2008)	12	73	16	151	6.0%	1.66 [0.74 , 3.72]
✕ Premi (2012)	12	94	15	200	0.0%	1.80 [0.81 , 4.03]
**Total (Wald[a])**		**3894**		**11456**	**100.0%**	**0.80 [0.65 , 0.98]**
Total events:	414		1578			

Test for overall effect: Z = 2.12 (P = 0.03)

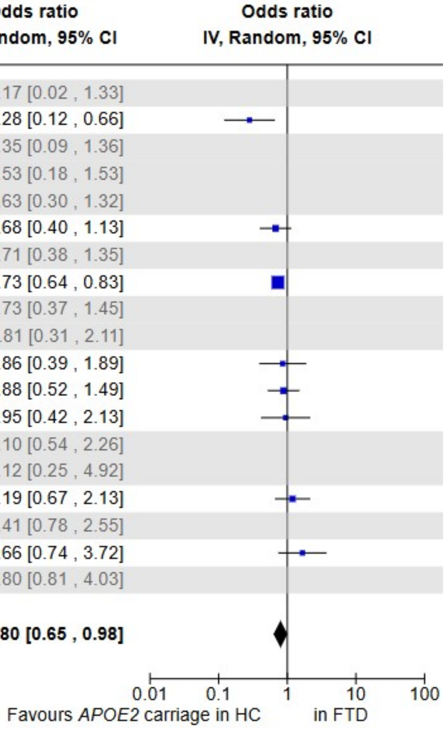

Heterogeneity: Tau² (REML[b]) = 0.02; Chi² = 12.41, df = 7 (P = 0.09); I² = 27%

Footnotes

[a]CI calculated by Wald-type method.

[b]Tau² calculated by Restricted Maximum-Likelihood method.

Figure S34 Exploratory meta-analysis of *APOE* ε2 carriage in frontotemporal dementia (FTD) vs. healthy controls (HC) among East Asian studies.

Study or Subgroup	Events	Total	Events	Total	Weight	Odds ratio IV, Random, 95% CI
✓ Kowalska (2001)	1	24	40	200	6.5%	0.17 [0.02 , 1.33]
✕ Bernardi (2006)	7	100	38	180	0.0%	0.28 [0.12 , 0.66]
✕ Agosta (2009)	3	31	13	56	0.0%	0.35 [0.09 , 1.36]
✓ Ji (2013)	4	62	44	381	23.7%	0.53 [0.18 , 1.53]
✕ Sadhukhan (2023)	10	122	32	256	0.0%	0.63 [0.30 , 1.32]
✕ Srinivasan (2006)	19	161	125	756	0.0%	0.68 [0.40 , 1.13]
✕ Rogalski (2011)	14	149	42	330	0.0%	0.71 [0.38 , 1.35]
✕ Mishra (2017)	307	3046	1250	9400	0.0%	0.73 [0.64 , 0.83]
✓ Chen (2016)	10	82	183	1149	57.7%	0.73 [0.37 , 1.45]
✕ Nielsen (2003)	6	49	24	163	0.0%	0.81 [0.31 , 2.11]
✕ Seripa (2011)	13	86	17	99	0.0%	0.86 [0.39 , 1.89]
✕ Seripa (2012)	29	282	34	296	0.0%	0.88 [0.52 , 1.49]
✕ Riemenschneider (2002)	9	52	33	182	0.0%	0.95 [0.42 , 2.13]
✕ Lovati (2010)	10	75	62	506	0.0%	1.10 [0.54 , 2.26]
✓ Wang (2020)	4	30	4	33	12.1%	1.12 [0.25 , 4.92]
✕ Verpillat (2002)	18	94	65	392	0.0%	1.19 [0.67 , 2.13]
✕ Daniele (2009)	19	127	38	343	0.0%	1.41 [0.78 , 2.55]
✕ Albani (2008)	12	73	16	151	0.0%	1.66 [0.74 , 3.72]
✕ Premi (2012)	12	94	15	200	0.0%	1.80 [0.81 , 4.03]
**Total (Wald[a])**		**198**		**1763**	**100.0%**	**0.65 [0.39 , 1.09]**
Total events:	19		271			

Test for overall effect: Z = 1.63 (P = 0.10)

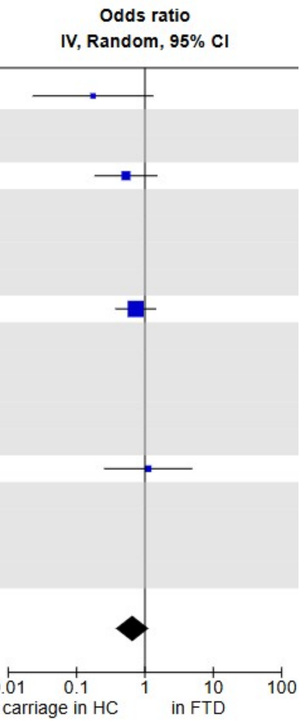

Heterogeneity: Tau² (REML[b]) = 0.00; Chi² = 2.39, df = 3 (P = 0.49); I² = 0%

Footnotes

[a]CI calculated by Wald-type method.

[b]Tau² calculated by Restricted Maximum-Likelihood method.

Figure S35 Exploratory meta-analysis of *APOE* ε2 carriage in frontotemporal dementia (FTD) vs. Alzheimer's disease (AD) among East Asian studies.

Study or Subgroup	Events	Total	Events	Total	Weight	Odds ratio IV, Random, 95% CI
✓ Kowalska (2001)	1	24	3	65	5.2%	0.90 [0.09 , 9.08]
✓ Ji (2013)	4	62	26	432	23.7%	1.08 [0.36 , 3.20]
✓ Chen (2016)	10	82	90	1026	57.9%	1.44 [0.72 , 2.90]
✕ Rogalski (2011)	14	149	11	179	0.0%	1.58 [0.70 , 3.60]
✕ Nielsen (2003)	6	49	21	320	0.0%	1.99 [0.76 , 5.20]
✕ Sadhukhan (2023)	10	122	4	94	0.0%	2.01 [0.61 , 6.62]
✓ Wang (2020)	4	30	4	63	13.1%	2.27 [0.53 , 9.78]
✕ Lovati (2010)	10	75	40	735	0.0%	2.67 [1.28 , 5.59]
✕ Agosta (2009)	3	31	1	51	0.0%	5.36 [0.53 , 53.97]
**Total (Wald[a])**		**198**		**1586**	**100.0%**	**1.39 [0.82 , 2.37]**
Total events:	19		123			

Test for overall effect: Z = 1.23 (P = 0.22)

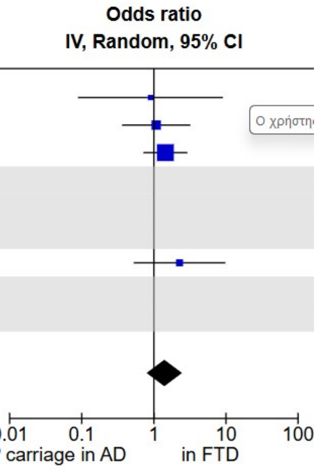

Heterogeneity: Tau^2 (REML[b]) = 0.00; Chi^2 = 0.79, df = 3 (P = 0.85); I^2 = 0%

Footnotes

[a]CI calculated by Wald-type method.

[b]Tau^2 calculated by Restricted Maximum-Likelihood method.
